# Discovery and engineering of Tsp2Cas9 for genome editing

**DOI:** 10.1038/s41421-024-00685-w

**Published:** 2024-05-21

**Authors:** Huilin Mao, Yuwen Tian, Ziwen Wang, Jingtong Liu, Jingjing Wei, Yao Wang, Chen Tao, Miaomiao Li, Shengzhou Wang, Li Shen, Junnan Tang, Rui Wang, Song Gao, Feng Lan, Yongming Wang

**Affiliations:** 1 Department of Cardiology, The First Affiliated Hospital of Zhengzhou University, Zhengzhou, Henan China; 2 Center for Medical Research and Innovation, Shanghai Pudong Hospital, Fudan University Pudong Medical Center, School of Life Sciences, Zhongshan Hospital, Human Phenome Institute, Shanghai Engineering Research Center of Industrial Microorganisms, Fudan University, Shanghai, China; 3 State Key Laboratory of Oncology in South China, Collaborative Innovation Center for Cancer Medicine, Sun Yat-sen University Cancer Center, Guangzhou, Guangdong China; 4 State Key Laboratory of Cardiovascular Disease, Fuwai Hospital, National Center for Cardiovascular Diseases, Chinese Academy of Medical Sciences and Peking Union Medical College, Beijing, China; 5 International Human Phenome Institutes, Shanghai, China

**Keywords:** Cell biology, DNA damage and repair

Dear Editor,

The CRISPR-Cas9 system stands as a potent genome-editing tool deployed across various organisms^1^. Within this system, a guide RNA (gRNA) collaborates with a Cas9 nuclease, forming a complex wherein the gRNA specifies the target site, and the Cas9 executes DNA cleavage^2^. Additionally, base editors have been developed by coupling a nickase form of Cas9 nuclease (Cas9n) with a deaminase, enabling base alterations at the target sites^3^. Recently, Cas9n was fused with an engineered reverse transcriptase (RT) enzyme to enable the development of prime editors capable of generating genomic changes over large stretches of base pairs^4^. However, a notable constraint of CRISPR-Cas9 system lies in its reliance on protospacer adjacent motif (PAM) sequences at target sites, limiting its scope of targeting^2^. Public databases harbour thousands of computationally identified Cas9 orthologs, each characterized by unique PAM requirements^5^. By developing new Cas9 genome-editing tools, it is becoming feasible to edit a broader array of genomic sites.

It has been demonstrated that phylogenetically closely related Cas9 orthologs (with > 50% amino acid identity) can share the same single guide RNA (sgRNA) scaffold^6^. In this study, we selected 15 sgRNAs that had been previously validated through in vitro cleavage assay^7,8^. For each sgRNA, we synthesized 3–15 human-codon-optimized Cas9 orthologs, resulting in 15 groups. We synthesized a total of 82 Cas9 orthologs, 9 belonging to type II-A and the remaining 73 belonging to type II-C (Supplementary Fig. S1a and Table S1).

The activity of each Cas9 ortholog was assessed by a previously developed GFP-activation assay^5^, where genome editing at the target site will induce GFP expression in human cells (Supplementary Fig. S1b). We tested each Cas9 activity using both 20- and 24-nt guide sequences. GFP-activation assay showed that two type II-A Cas9 orthologs (LcoCas9 and Tsp2Cas9) and three types II-C Cas9 orthologs (BcaCas9, Cci2Cas9, and CspCas9) were active in human cells (Supplementary Fig. S1c), accounting for 6% of all tested Cas9 orthologs. These data suggest that only a small subset of Cas9 orthologs are active in mammalian cells.

Next, we analyzed PAMs for these five active Cas9 orthologs. The GFP-positive cells were sorted by flow cytometry, and the 8-bp random sequence was PCR-amplified for deep sequencing. The sequencing results showed that these Cas9 orthologs generated indels at the target sites (Supplementary Fig. S2a). We constructed the WebLogo diagram (Supplementary Fig. S2b) and the PAM wheel (Supplementary Fig. S2c) for each Cas9 based on deep sequencing data. The results showed that all these Cas9 orthologs recognized degenerate PAMs. BcaCas9 preferred an NRRNS PAM (R = A or G; S = C or G); Cci2Cas9 preferred an NNRNMC PAM (M = A or C); CspCas9 preferred an NRVY PAM (V = A or C or G; Y = C or T); LcoCas9 and Tsp2Cas9 PAMs preferred an NNRRR PAM. We shall focus on Tsp2Cas9 in the following study.

**Fig. 1:**
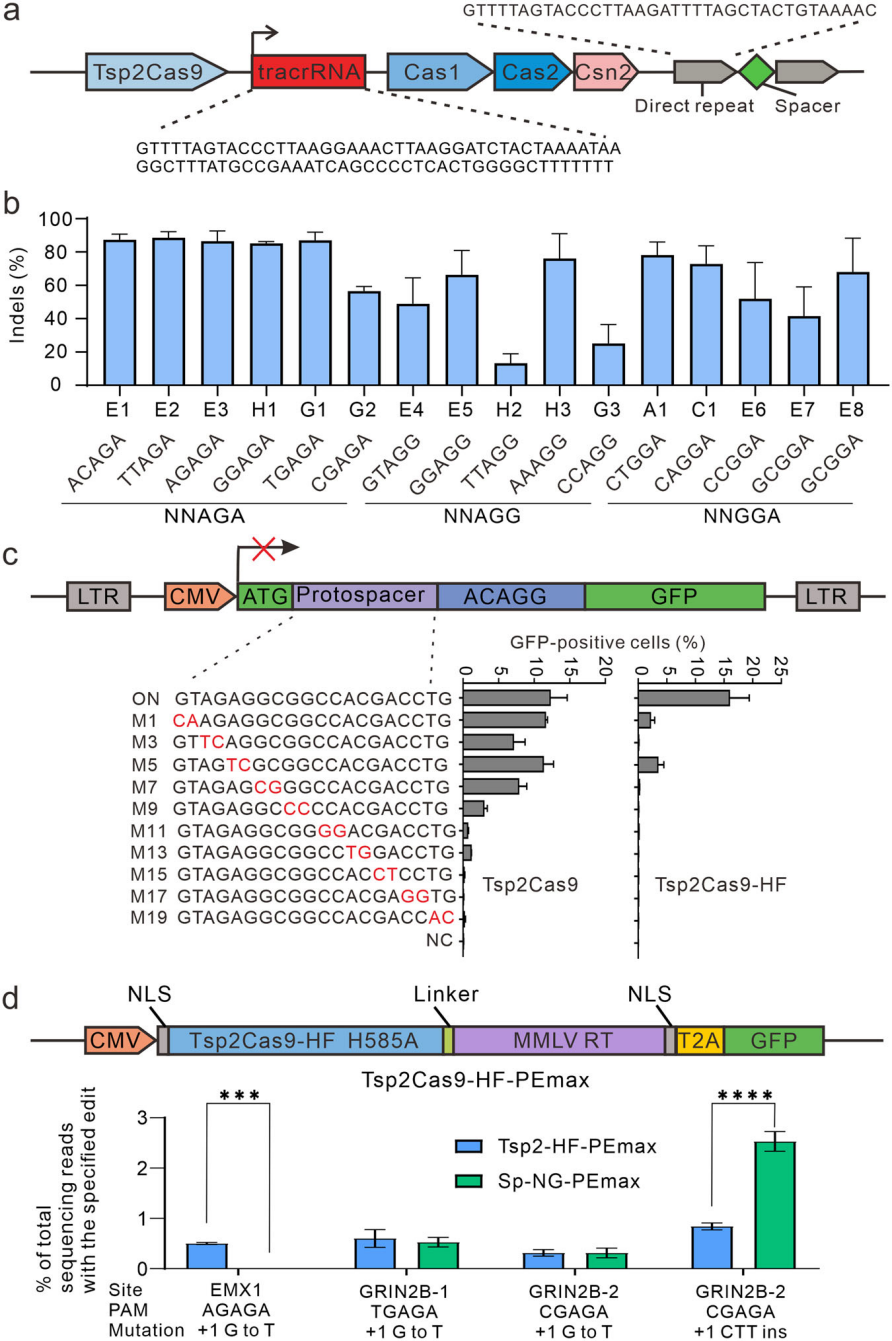
Genome-editing with Tsp2Cas9. **a** Genetic organization of the *CRISPR-Tsp2Cas9* locus. Direct repeat and tracrRNA sequences are shown. **b** Tsp2Cas9 enables genome-editing at a panel of 16 endogenous loci (*n* = 3). **c** Analysis of Tsp2Cas9 and Tsp2Cas9-HF specificity with GFP-activation assay. Schematic illustration of GFP-activation assay (top). sgRNAs with dinucleotide mutations and their activity (bottom). **d** Prime editing with Tsp2Cas9-HF-PEmax. Schematic illustration of the Tsp2Cas9-HF-based prime editor (top). SpCas9-NG-PEmax is used for comparison (*n* = 3). Mutation types are shown below. A value of *P* < 0.05 was considered significant (***P < 0.001, **** *P* < 0.0001).

The Tsp2Cas9 locus consists of three Cas genes (i.e. Cas9, Cas1, Cas2), 12 repeat sequences, and a tracrRNA (Fig. 1a). Tsp2Cas9 had 47.6% sequence identity with St1Cas9. To test whether Tsp2Cas9 enabled mammalian genome editing, we selected 16 endogenous targets with NNAGA, NNAGG, and NNGGA PAMs across 5 genes. We transfected Tsp2Cas9 and sgRNA expression plasmids into HEK293T cells, followed by puromycin selection. The results showed that Tsp2Cas9 enabled efficient genome editing at these loci (Fig. 1b). We tested Tsp2Cas9 in additional cell lines, including HeLa, SH-SY5Y, K562, and a mouse cell line (N2a) and achieved efficient genome editing in all the cell lines (Supplementary Fig. S3a–d).

We further compared activity of Tsp2Cas9 to that of the canonical SpCas9 at a panel of 17 endogenous loci without drug selection. Overall, SpCas9 demonstrated superior efficiency (Supplementary Fig. S4a, b). However, it is noteworthy that Tsp2Cas9 exhibited comparable or higher activity at 7 of the loci.

Next, we analyzed Tsp2Cas9 specificity by GFP-activation assay. We designed a panel of 10 sgRNAs with dinucleotide mutations along the protospacer. The on-target sgRNA was used as a control. The results showed that Tsp2Cas9 more tolerated mismatches at the PAM-distal region and less tolerated mismatches at the PAM-proximal region (Fig. 1c). Previous studies have shown that Cas9 residues can form hydrogen bonds to phosphate backbone of the target DNA strand, and disruption of these hydrogen bonds can increase specificity^9^. To improve specificity, we analyzed the St1Cas9–sgRNA crystal structure^10^ and identified 11 residues that can form hydrogen bonds (Supplementary Fig. S5a). We aligned Tsp2Cas9 to St1Cas9 and identified corresponding residues that potentially form hydrogen bonds (Supplementary Fig. S5b).

Next, we replaced the individual residue with alanine and tested specificity of each variant. We designed 3 sgRNAs with dinucleotide mutations at the PAM-distal region. The results showed that all mutations could improve specificity (Supplementary Fig. S6a, b). Since R261A and N434A mutations had more specificity without decreasing activity, we further tested them by designing a panel of 10 sgRNAs with dinucleotide mutations. We also tested the G251A mutation, which was close to the R261A mutation. The results showed that the R261A mutation performed better than the G251A and N434A mutations (Supplementary Fig. S7a). When R261A was combined with either N434A, G251A, or both, the specificity was further improved (Supplementary Fig. S7b). We named Tsp2Cas9-G251A/R261A/N434A as Tsp2Cas9-HF, which displayed the highest specificity (Fig. 1c).

Next, we performed genome-wide unbiased identification of double-stranded breaks enabled by sequencing (GUIDE-seq)^11^ to test Tsp2Cas9-HF specificity in HEK293T cells. We designed 4 targets on *AAVS1*, *EMX1*, and *GRIN2B* genes. The sequencing results revealed high on-target efficiency (Supplementary Fig. S8a–d). Tsp2Cas9 induced a total of 11 off-targets, while Tsp2Cas9-HF induced one off-target. To verify the off-target sites, we selected 4 off-target sites for the *GRIN2B-sg1* locus and performed targeted deep sequencing. The results showed that indels occurred at these off-target sites (Supplementary Fig. S8e).

We further compared specificity of Tsp2Cas9-HF with two SpCas9 variants, SpCas9-NG^12^ and SpCas9-HF1^9^, at 3 loci using GUIDE-seq. For the *AAVS1* locus, Tsp2Cas9-HF, SpCas9-NG, and SpCas9-HF1 indued 0, 1, and 21 off-targets, respectively (Supplementary Fig. S9a). For the *EMX1* locus, Tsp2Cas9-HF, SpCas9-NG, and SpCas9-HF1 indued 0, 2, and 26 off-targets, respectively (Supplementary Fig. S9b). For the *GRIN2B* locus, Tsp2Cas9-HF, SpCas9-NG, and SpCas9-HF1 indued 0, 0, and 36 off-targets, respectively (Supplementary Fig. S9c). These results demonstrated the high specificity of Tsp2Cas9-HF and SpCas9-NG.

To elucidate the impact of G251A, R261A, and N434A mutations on Tsp2Cas9 specificity, we constructed a SWISS-MODEL of Tsp2Cas9 using the St1Cas9 crystal structure as a template (Supplementary Fig. S10). The resulting structure revealed that upon mutating G251/R261/N434, the capacity of Cas9 to form hydrogen bonds with dsDNA was lost, diminishing the strength of interaction with dsDNA and impeding the binding of mismatched dsDNA, thereby reducing off-target effects.

We compared the activity of Tsp2Cas9-HF to that of wild-type Tsp2Cas9 in HEK293T cells. They achieved similar protein expression levels (Supplementary Fig. S11a, b). We designed a panel of 10 endogenous targets with NNAGA and NNAGG PAMs. Interestingly, Tsp2Cas9-HF achieved similar editing efficiencies to wild-type Tsp2Cas9 (Supplementary Fig. S11c, d). These data demonstrated that mutations in Tsp2Cas9-HF did not disrupt activity.

Next, we compared the activity of Tsp2Cas9-HF to those of SpCas9-NG and SpCas9-HF1. Both the latter variants were cloned into the Tsp2Cas9-HF expression construct (Supplementary Fig. S12a). We designed a penal of 12 endogenous targets with compatible PAMs for these Cas9 nucleases. The editing results showed that Tsp2Cas9-HF achieved lower editing efficiencies than SpCas9-NG and SpCas9-HF1 (Supplementary Fig. S12b, c).

Next, we tested whether Tsp2Cas9-HF could be used for prime editing. We aligned Tsp2Cas9-HF to St1Cas9 to identify the conserved catalytic residue H585 within the HNH nuclease domain. Tsp2Cas9-HF H585A was expected to nick the PAM-containing strand. We used Tsp2Cas9-HF H585A to replace SpCas9 H840A in the PEmax prime editor^13^, resulting in a new prime editor named Tsp2Cas9-HF-PEmax (Fig. 1d). Meanwhile, we also used SpCas9-NG H840A to replace SpCas9 H840A of PEmax, resulting in SpCas9-NG-PEmax for comparison. We selected 3 endogenous sites and designed prime editing guide RNAs (pegRNAs) to replace a G with a T at these sites. The results showed that Tsp2Cas9-HF-PEmax displayed higher efficiency than SpCas9-NG-PEmax at the *EMX1* locus (Fig. 1d; Supplementary Fig. S13a–c). Tsp2Cas9-HF-PEmax displayed comparable efficiency to SpCas9-NG-PEmax at the other 2 loci targeting *GRIN2B* gene (Fig. 1d). Moreover, when a pegRNA was designed to insert a CTT at the *GRIN2B-2* locus, SpCas9-NG-PEmax displayed higher efficiency than Tsp2Cas9-HF-PEmax (Fig. 1d; Supplementary Fig. S13d).

We further designed pegRNAs containing 10 distinct mutation types targeting the *EMX1* locus and 10 mutation types targeting the *GRIN2B* locus (Supplementary Table S2). Notably, SpCas9-NG-PEmax successfully induced three types of desired mutations, while Tsp2Cas9-HF-PEmax failed to generate any desired mutations (Supplementary Fig. S14a, b). No editing events were detected in HeLa and SH-SY5Y cells with Tsp2Cas9-HF-PEmax (Supplementary Fig. S15a, b). These results highlight the need for further optimization of Tsp2Cas9-HF-PEmax to enhance its efficacy for future applications.

Recently, a novel PE6d prime editor with a reduced size and enhanced activity has been developed^14^. Considering this advancement, we constructed Tsp2Cas9-HF-PE6d and SpCas9-NG-PE6d variants and evaluated their performance with the aforementioned 10 pegRNAs. SpCas9-NG-PE6d exhibited efficiency comparable to SpCas9-NG-PEmax, generating three types of desired mutations, whereas Tsp2Cas9-HF-PE6d failed to produce any desired mutations (Supplementary Fig. S16a, b).

Then we explored the potential of Tsp2Cas9-HF for base editing applications. We replaced the nickase used in ABEmax by Tsp2Cas9-HF and developed Tsp2Cas9-HF-ABEmax. We observed promising results when targeting 3 genes using a panel of 6 sgRNAs. Tsp2Cas9-HF-ABEmax achieved 48.4% and 8.0% A to G conversion efficiency at the *E1-A5* and *E2-A6* loci, respectively (Supplementary Fig. S17a, b). However, conversion efficiency remained low (< 5%) at the remaining loci. These findings underscore the necessity for further refining of Tsp2Cas9-HF-ABEmax.

In summary, we successfully identified a compact Tsp2Cas9 as an efficient genome-editing tool after screening 82 Cas9 orthologs. Moreover, we engineered Tsp2Cas9 to improve its specificity. Importantly, we demonstrated the potential of Tsp2Cas9 for prime editing and base editing. With further development, new genome-editing tools will expand the capabilities of genome editing.

### Supplementary information

Supplementary information

Supplementary tables
