# Supplementary material for: Discovery and engineering of Tsp2Cas9 for genome editing
Source: Cell Discov. 2024 May 21;10:55. doi: 10.1038/s41421-024-00685-w (PMC11106062; doi:10.1038/s41421-024-00685-w)
Supplement: Supplementary file 1 — Supplementary information [file 41421_2024_685_MOESM1_ESM.pdf]

Discovery and engineering of Tsp2Cas9 for genome editing

Huilin Mao[1,2], Yuwen Tian[2], Ziwen Wang[3], Jingtong Liu[2], Jingjing Wei[2], Yao Wang[2], Chen Tao[2], Miaomiao Li[2], Shengzhou Wang[2], Li Shen[2], Junnan Tang[1], Rui Wang[2], Song Gao[3], Feng Lan[4], Yongming Wang[1,2,5]

[1]Department of Cardiology, The First Affiliated Hospital of Zhengzhou University, Zhengzhou, Henan, China. [2]Center for Medical Research and Innovation, Shanghai Pudong Hospital, Fudan University Pudong Medical Center, School of Life Sciences, Zhongshan Hospital, Human Phenome Institute, Shanghai Engineering Research Center of Industrial Microorganisms, Fudan University, Shanghai, China. [3]State Key Laboratory of Oncology in South China, Collaborative Innovation Center for Cancer Medicine, Sun Yat-sen University Cancer Center, Guangzhou, Guangdong, China. [4]State Key Laboratory of Cardiovascular Disease, Fuwai Hospital, National Center for Cardiovascular Diseases, Chinese Academy of Medical Sciences and Peking Union Medical College, Beijing, China. [5]International Human Phenome Institutes, Shanghai, China.

These authors contributed equally: Huilin Mao, Yuwen Tian, Ziwen Wang

Correspondence: Yongming Wang (ymw@fudan.edu.cn), Feng Lan (fenglan@ccmu.edu.cn), Song Gao (gaosong@sysucc.org.cn), Rui Wang (wangr@fudan.edu.cn)

MATERIAL AND METHODS

Cell culture and transfection

HEK293T, Hela, SH-SY5Y, and N2a cells were maintained in DMEM (Gibco) supplemented with 10% FBS (Gibco). All the cells were supplemented with 100 U/ml penicillin and 100 mg/ml streptomycin and cultured at 37◦C and 5% CO2. HEK293T cells were transfected with Lipofectamine 2000 (Invitrogen) according to the manufacturer's instructions. For Cas9 PAM sequence screening, HEK293T cells were plated into 10 cm dishes and transfected at 50–60% confluency with a total of 10 μg of Cas9 plasmid and 5 μg of sgRNA plasmid in 10 cm dishes. For genome editing comparisons of Cas9, cells were plated into 24-well plates and transfected at 50–60% confluency with a total of 500 ng of Cas9 plasmid and 300 ng of sgRNA plasmid. For genome editing comparisons of PE, Cells were seeded in 12-well plates and transfected at approximately 60% confluence using Lipofectamine 2000 (Invitrogen) according to the manufacturer's protocols. A total of 1.5 μg PEmax-T2A-GFP and 500 ng pegRNA expression plasmids were co-transfected into HEK293T. 72 h after transfection, GFP-positive cells were collected from flow cytometry (MoFlo XDP flow cytometer) [1]. The genomic DNA of cells was extracted using QuickExtract DNA Extraction Solution (Epicentre) according to the manufacturer's protocols.

Plasmid construction

Cas9 expression plasmid construction: The plasmid pX459 (Addgene #62988) was amplified by the primers px459-F/px459-R to obtain the pX459 backbone. The human codon-optimized Cas9 gene (Table S1) was synthesized by HuaGene (Shanghai, China) and cloned into the pX459 backbone by the NEBuilder assembly tool (NEB) according to the manufacturer's instructions. Sequences of each Cas9 were confirmed by Sanger sequencing (GENEWIZ,

Suzhou, China).

sgRNA expression plasmid construction: sgRNA expression plasmids were constructed by ligating sgRNA into the BbsI-digested mU6-Tsp_tracr plasmid or the BsmBI-digested hU6-sp_tracr plasmid, respectively. The target sequences and primer sequences are listed in Tables S2 and S3, respectively.

PEmax expression plasmid construction: Firstly, T2A-GFP was inserted at the C terminal of pCMV- PE2(Addgene plasmid # 132775), resulting in pCMV-PE2-T2A-GFP. pCMV-Tsp2Cas9-HF-PEmax-T2A-GFP and pCMV-SpCas9-NG-PEmax-T2A-GFP were generated through Gibson assembly [2] by combining Tsp2Cas9-HF(H585A) (Sequence S1) or SpCas9-NG (H840A)(Sequence S2) [3], a linker and the codon-optimized M-MLV RT synthesized by HuaGene (Shanghai, China) with additional NLS sequences by PCR and inserting them into a pCMV-PE2-T2A-GFP backbone.

PE6d expression plasmid construction: pCMV-Tsp2Cas9-HF-PE6d-T2A-GFP(Sequence S3) and pCMV-SpCas9-NG-PE6d-T2A-GFP(Sequence S4) were generated through Gibson assembly by combining M-MLV RT which had T128N, V223Y, D200C mutation and RNaseH domain truncation[4] and the sequences from pCMV-Tsp2Cas9-HF-PEmax-T2A-GFP and pCMV- SpCas9-NG -PEmax-T2A-GFP backbone without M-MLV RT by PCR.

pegRNA expression plasmid construction: To generate pegRNA expression plasmids, PCR products, including spacer sequences, scaffold sequences, and 3' extension sequences, were amplified with indicated primers (Table S3) using Q5 High-Fidelity enzyme (NewEnglandBioLabs), which were subsequently cloned into the BsaI-digested pU6-pegRNA GG-acceptor [5]. Sequences of all pegRNA are listed in Table S2.

Flow cytometry analysis

Transfected library cells with a certain percentage of GFP-positive cells were collected by centrifugation at 1000 rpm for 5 min and resuspended in PBS. Then, GFP-positive cells were collected by flow cytometry and cultured in six-well plates. Five days after culture, the genomic DNA was isolated for deep sequencing.

PAM sequence analysis

Twenty base-pair sequences (AAGCCTTGTTTGCCACCATG/ GTGAGCAAGGGCGAGGAGCT) flanking the target sequence (GAACGGCTCGGAGATCATCATTGCGNNNNNNNN) were used to fix these two target sequences. CTG and GTGAGCAAGGGCGAGGAGCT were used to fix 8-bp random sequences. Target sequences with in-frame mutations were used for PAM analysis. The 8-bp random sequences were extracted and visualized by WebLogo [6] and a PAM wheel chart to identify PAMs [7].

Genome editing for endogenous sites

HEK293T, Hela, SH-SY5Y, and N2a cells were seeded into 24-well plates and transfected with a total of 500 ng of Cas9 plasmid and 300 ng of sgRNA plasmid by Lipofectamine 2000 (1.6 µL). The transfected HEK293T, Hela, SH-SY5Y, and N2a cells were selected using media supplemented with 1, 1, 0.5, and 2 µg/mL of puromycin, respectively. Cells were collected Three days after transfection and selection. The genomic DNA was isolated, and the target sites were PCR amplified and extracted by QuickExtract DNA Extraction Solution (Epicentre) for deep sequencing to check the editing efficiency. For genome editing comparisons of PE, 72 h after transfection, GFP-positive cells were collected from flow cytometry (MoFlo XDP flow cytometer). The genomic DNA was isolated, and the target sites were PCR amplified and extracted by QuickExtract DNA Extraction Solution (Epicentre) for deep sequencing to check the editing efficiency.

Test of Cas9 specificity

To test the specificity of Cas9, we generated GFP reporter cell lines with the ACAGG PAM. The cells were seeded into 48-well plates and transfected with 500 ng of Cas9 plasmids and 300 ng of sgRNA plasmids by using Lipofectamine 2000(1.6 μL). Five days after editing, the GFP-positive cells were analyzed using a Calibur instrument (BD). The data were analyzed using FlowJo.

GUIDE-seq

GUIDE-seq experiments were performed as described previously [8], with minor modifications. Firstly, 2 × 10^5 HEK293T cells were transfected with 500 ng of Cas9 plasmid, 500 ng of sgRNA plasmids, and 100 pmol of annealed GUIDE-seq oligonucleotides by electroporation and then seeded into 12-wells plate. The electroporation voltage, width, and number of pulses were 1,150 V, 30 ms, and two pulses, respectively. Genomic DNA was extracted with the DNeasy Blood and Tissue kit (QIAGEN) 6 days after electroporation according to the manufacturer's protocol. The genome library was prepared and subjected to deep sequencing [8]. For data analysis, GUIDE-seq (v1.02, https://github.com/aryeelab/guideseq) was performed using Python 3.9. The BWA-MEM algorithm with default parameters was utilized to align fastq files. During the identification step, reads were filtered if the mapping quality was <50 or if the number of mismatch sites exceeded 8. Identified off-targets were visualized without applying a background filter during the visualization step.

Western blotting

One day before transfection, HEK293T cells were seeded into a 6-well plate. For each well, 2 μg of Cas9-expressing plasmid were transfected using 4 μL of Lipofectamine2000. Three days after transfection, cell samples were collected, and total proteins were extracted using NP-40 buffer (Beyotime) supplemented

with 1 mM phenylmethanesulfonyl fluoride (PMSF) (Beyotime). The protein was separated by SDS-PAGE gel and transferred onto a polyvinylidene fluoride (PVDF) (Thermo) membrane. After transfer, the membrane was blocked with 5% (wt./ vol.) BSA (Sigma) in TBS-T (0.1% Tween 20 in 1× TBS) buffer and then incubated in the primary antibody (Anti-DDDDK tag (1:1,000; ab205606, Abcam) and anti-GAPDH (1:2,000; 5174s, Cell Signaling) at 4˚C overnight. Wash the membrane three times in TBS-T for 10 min each time. The second antibody (1:10,000; ab6721, Abcam) was incubated for 1 h at room temperature and then washed three times and imaged.

Statistical analysis

All the data are shown as mean ± SD. Statistical analyses were performed using Microsoft Excel. Two-tailed, paired Student t-tests were used to determine statistical significance when comparing two groups, whereas analyses of variance (ANOVAs) are used for comparisons between three or more groups. A value of $P < 0.05$ was considered to be statistically significant (*$P < 0.05$, **$P < 0.01$, ***$P < 0.001$, **** $P < 0.0001$).

REFERENCES

1 Feng, Y. *et al.* Enhancing prime editing efficiency and flexibility with tethered and split pegRNAs. *Protein & Cell* **14**, 304-308, doi:10.1093/procel/pwac014 (2023).

2 Chen, P. J. *et al.* Enhanced prime editing systems by manipulating cellular determinants of editing outcomes. *Cell* **184**, 5635-5652 e5629, doi:10.1016/j.cell.2021.09.018 (2021).

3 Wu, S. *et al.* Two Compact Cas9 Ortholog-Based Cytosine Base Editors Expand the DNA Targeting Scope and Applications In Vitro and In Vivo. *Front Cell Dev Biol* **10**, 809922, doi:10.3389/fcell.2022.809922 (2022).

4 Doman, J. L. *et al.* Phage-assisted evolution and protein engineering yield compact, efficient prime editors. *Cell* **186**, 3983-4002.e3926,

doi:10.1016/j.cell.2023.07.039 (2023).

5 Anzalone, A. V. *et al.* Search-and-replace genome editing without double-strand breaks or donor DNA. *Nature* **576**, 149-157, doi:10.1038/s41586-019-1711-4 (2019).

6 Crooks, G. E., Hon, G., Chandonia, J. M. & Brenner, S. E. WebLogo: A sequence logo generator - eScholarship. *Genome Research* **14**, 1188-1190 (2004).

7 Leenay, R. *et al.* Identifying and Visualizing Functional PAM Diversity across CRISPR-Cas Systems. *Molecular Cell* **62**, 137-147 (2016).

8 Tsai, S. Q. *et al.* GUIDE-seq enables genome-wide profiling of off-target cleavage by CRISPR-Cas nucleases. *Nat Biotechnol* **33**, 187-197, doi:10.1038/nbt.3117 (2015).

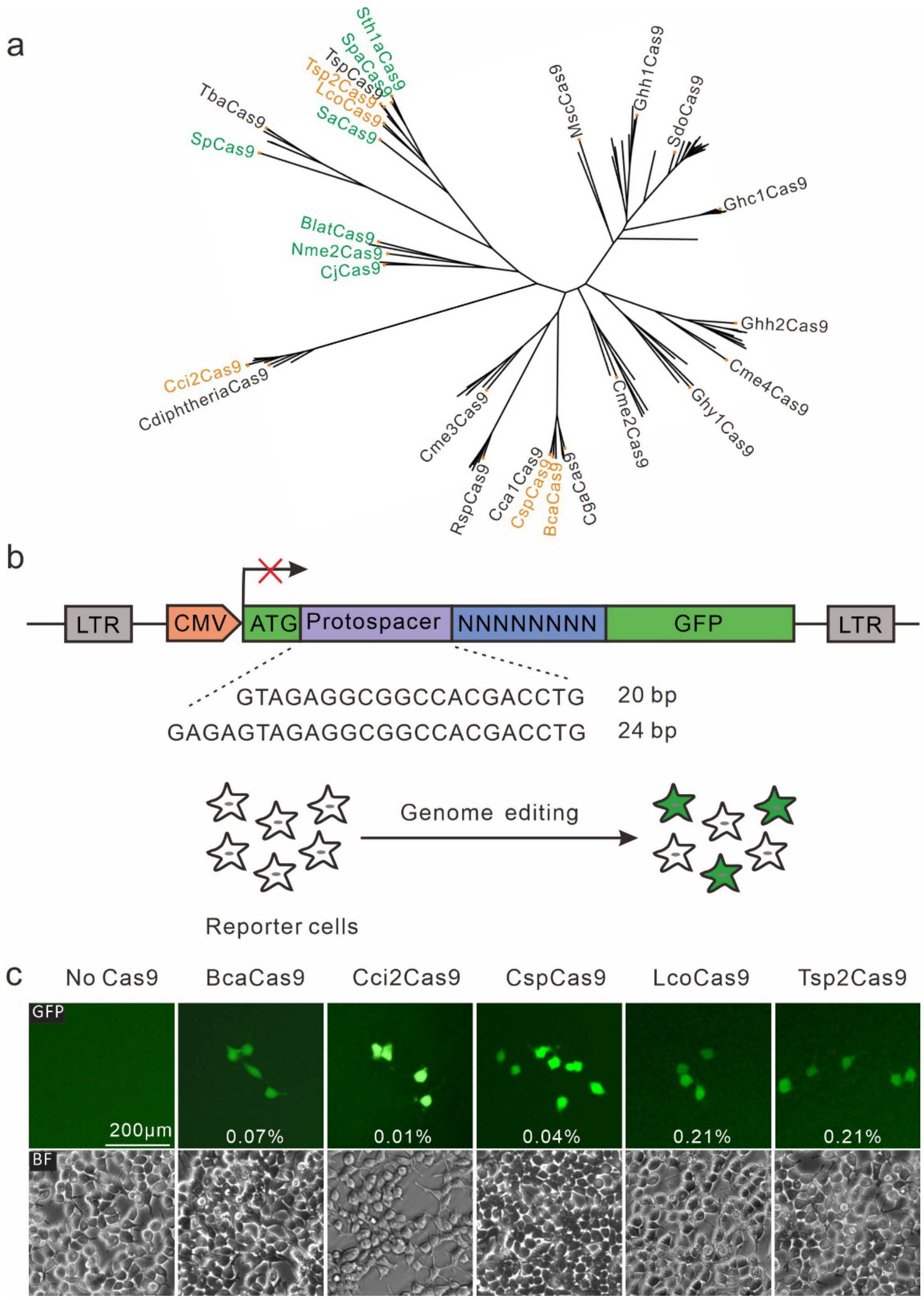

Supplementary Fig. S1 A screen of Cas9 activity. (A) Phylogenetical analysis of Cas9s. Cas9 activity confirmed by in vitro cleavage assay is shown in black. Cas9 activity previously confirmed in mammalian cells is shown in black. Cas9 activity confirmed in this study is shown in yellow. (B) Schematic of the GFP-activation assay. A protospacer followed by 8-bp random sequences is inserted into a GFP gene, resulting in a frameshift

mutation. Genome editing leads to GFP expression in a portion of cells. Protospacer sequences are shown below. (C) Genome editing results in GFP expression. The percentage of GFP-positive cells is shown in the picture.

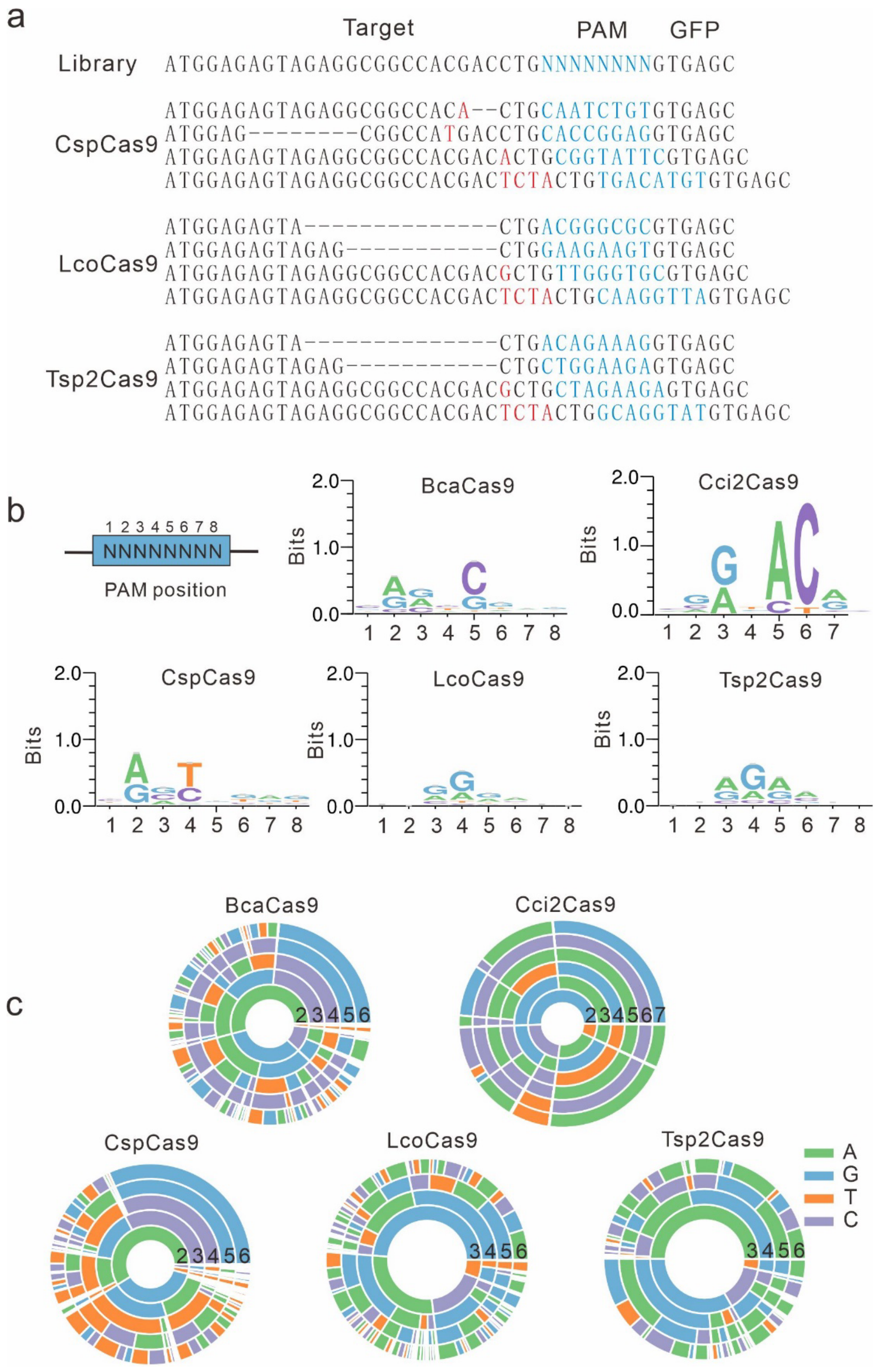

Supplementary Fig. S2 Analysis of PAMs. (A) The deep sequence shows that indels occur at the protospacer region in the GFP-activation assay. PAMs are shown in blue. Insertions are shown in red. (B) PAM logos are constructed based on deep sequencing data. (C) PAM wheels are constructed based on deep sequencing data.

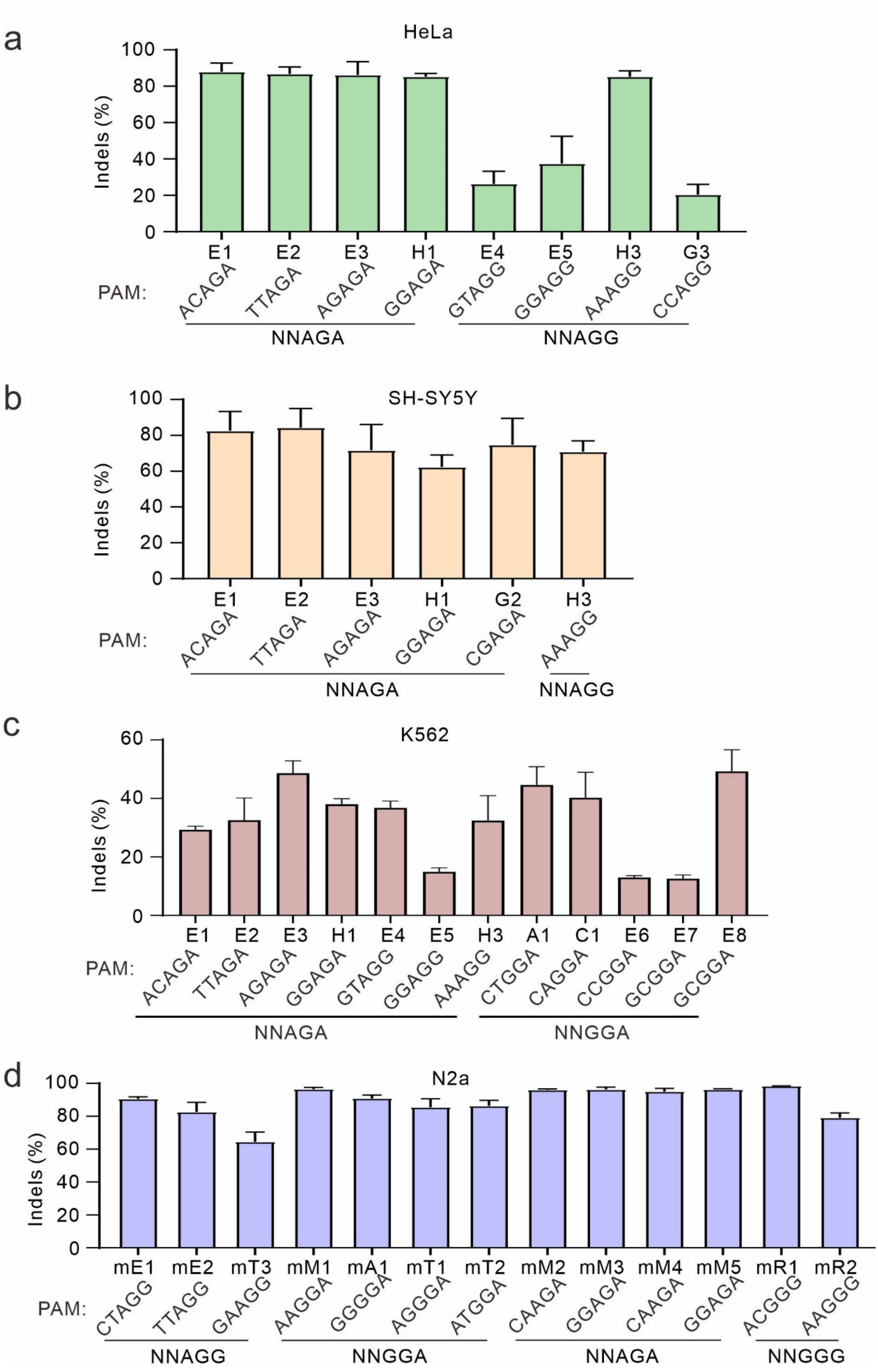

Supplementary Fig. S3: Tsp2Cas9 Enables Genome Editing in Various

Cell Lines. Tsp2Cas9 facilitates genome editing in HeLa cells (A), SH-SY5Y cells (B), K562 cells (C), and mouse N2a cells (D). The designations E1-8 are located in the EMX1 gene; G2 and G3 in the GRIN2B gene; H1 in the HGH1 gene; H3 in the HEK3 gene; A1 in the AAVS1 locus; C1 in the COMMD2 gene; mE1 and mE2 in the mouse EMX1 gene; mM1, mM2, mM3, mM4, and mM5 in the mouse Myh6 gene; mA1 in the mouse Apob gene; mR1 and mR2 in the mouse RNF2 gene; mT1, mT2, and mT3 derived from the mouse Th gene.

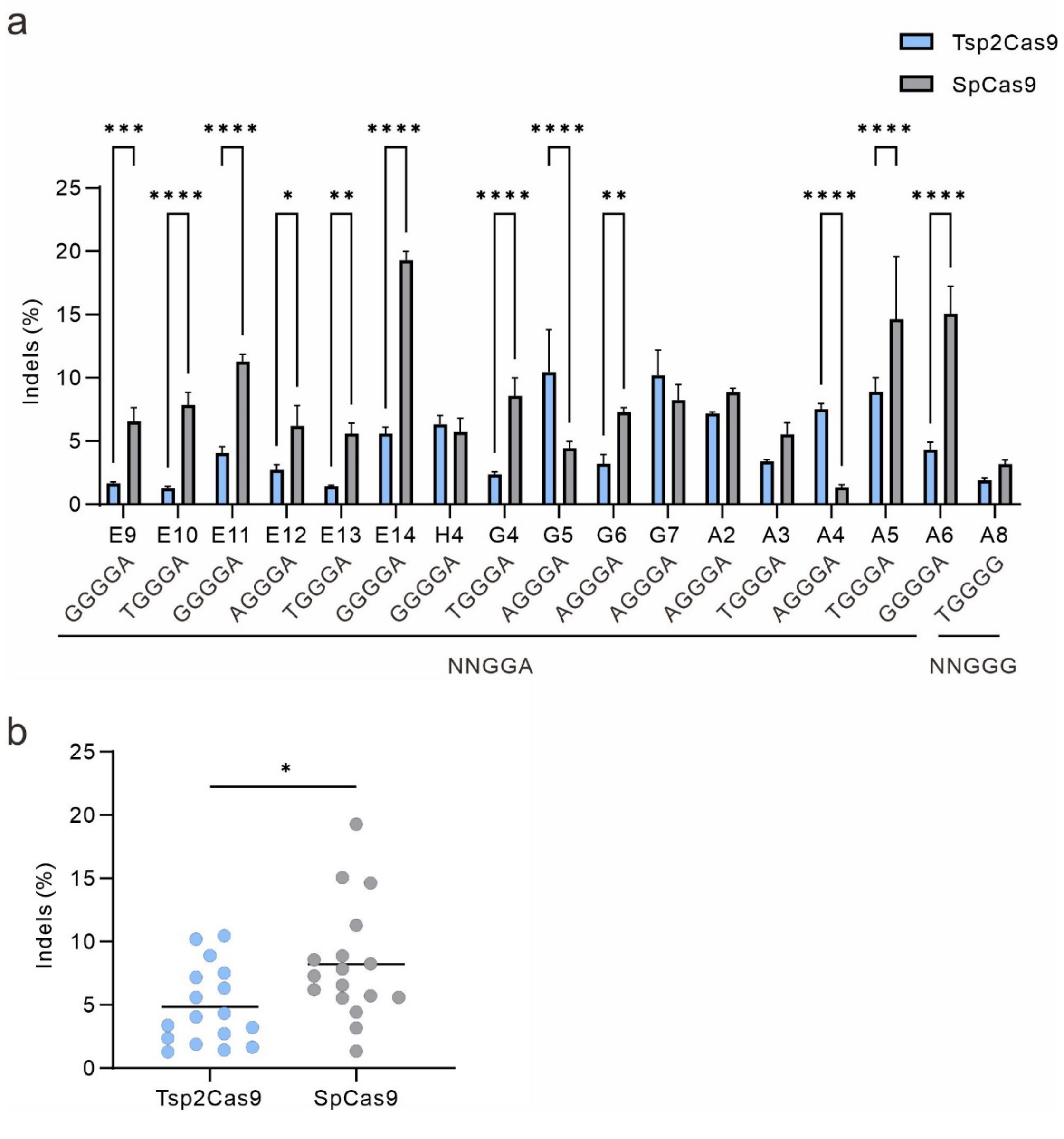

Supplementary Fig. S4: Comparison of activity between Tsp2Cas9 and SpCas9. (A) Genome editing efficiency comparison of Tsp2Cas9 and SpCas9 across a panel of 17 endogenous loci. (B) Quantification of editing efficiency for Tsp2Cas9 and SpCas9.

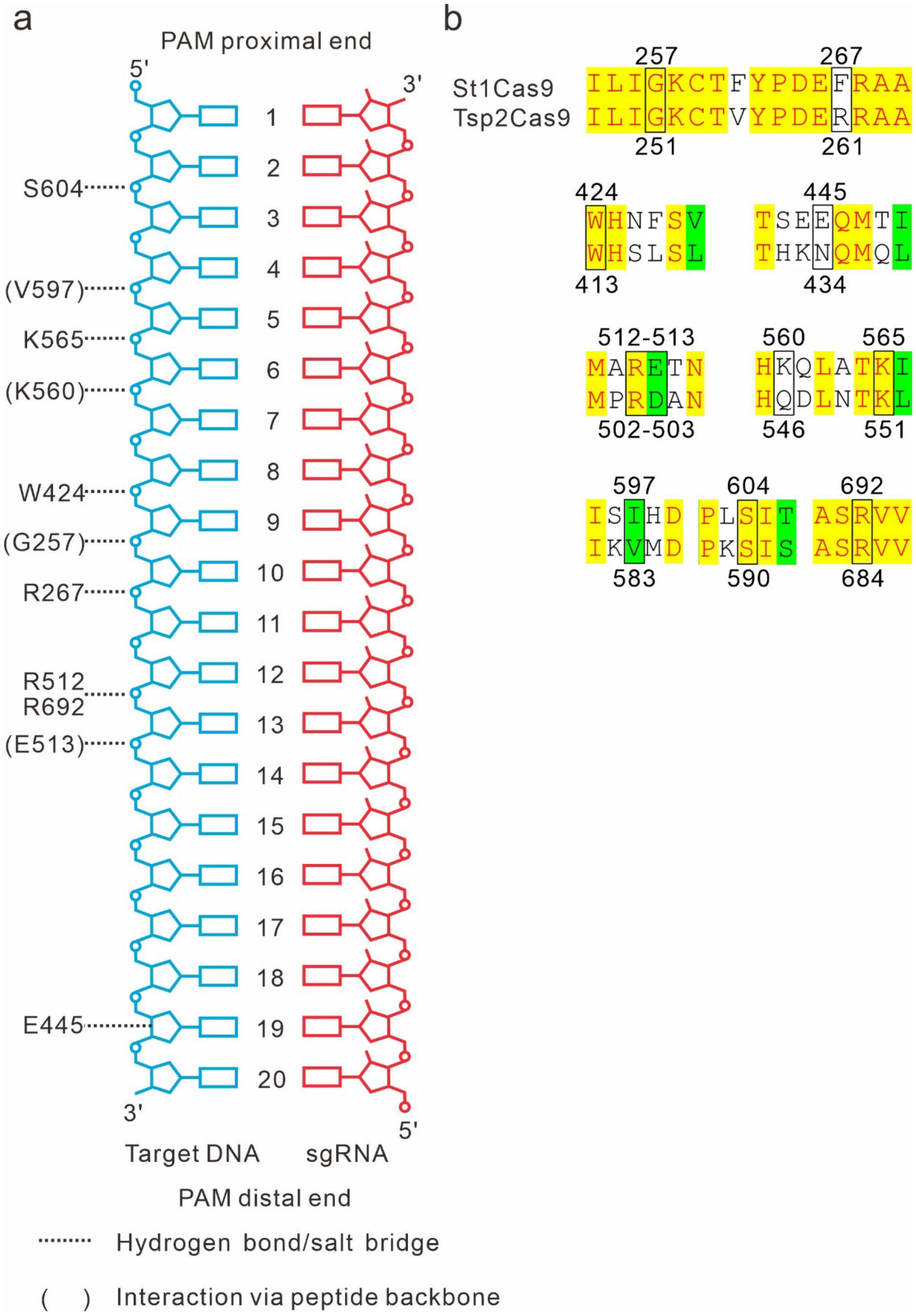

Supplementary Fig. S5 Analysis of Tsp2Cas9 residues potentially forming hydrogen bonds to the target DNA. (A) Schematic predicting St1Cas9 interactions with the target DNA–sgRNA duplex. (B) Protein sequence alignment of Tsp2Cas9 with St1Cas9.

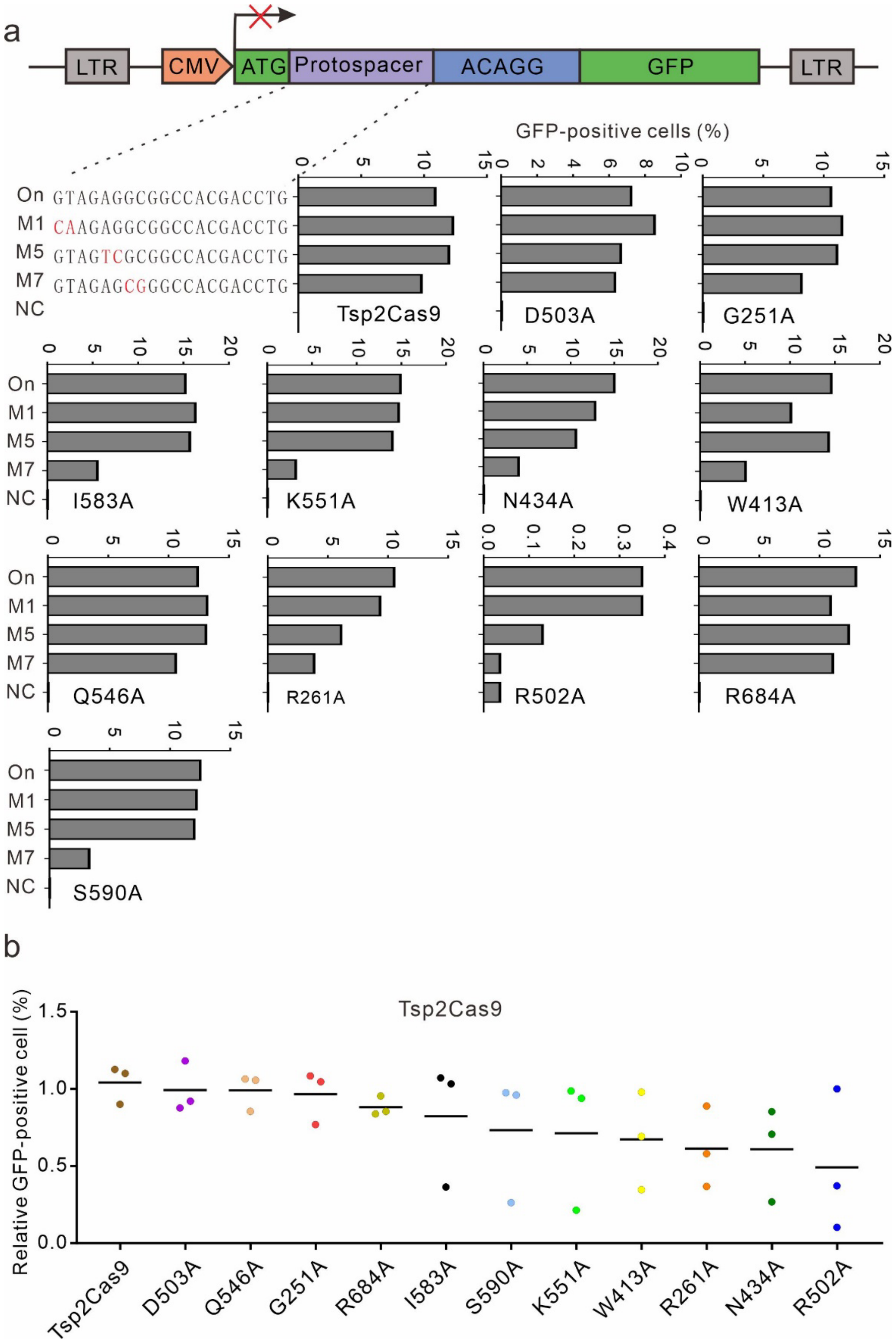

Supplementary Fig. S6 Analysis of each mutation on Tsp2Cas9 specificity with the GFP-activation assay. (A)Three sgRNAs with dinucleotide mutations are used to evaluate specificity. A Schematic of the

GFP-activation assay is shown above. sgRNAs and their activity are shown below. (B) Comparison of editing efficiency at off-targets based on GFP-activation assay among wild-type Tsp2Cas9 and 11 variants in HEK293T cells. The editing efficiency at off-targets is normalized by on-target efficiency.

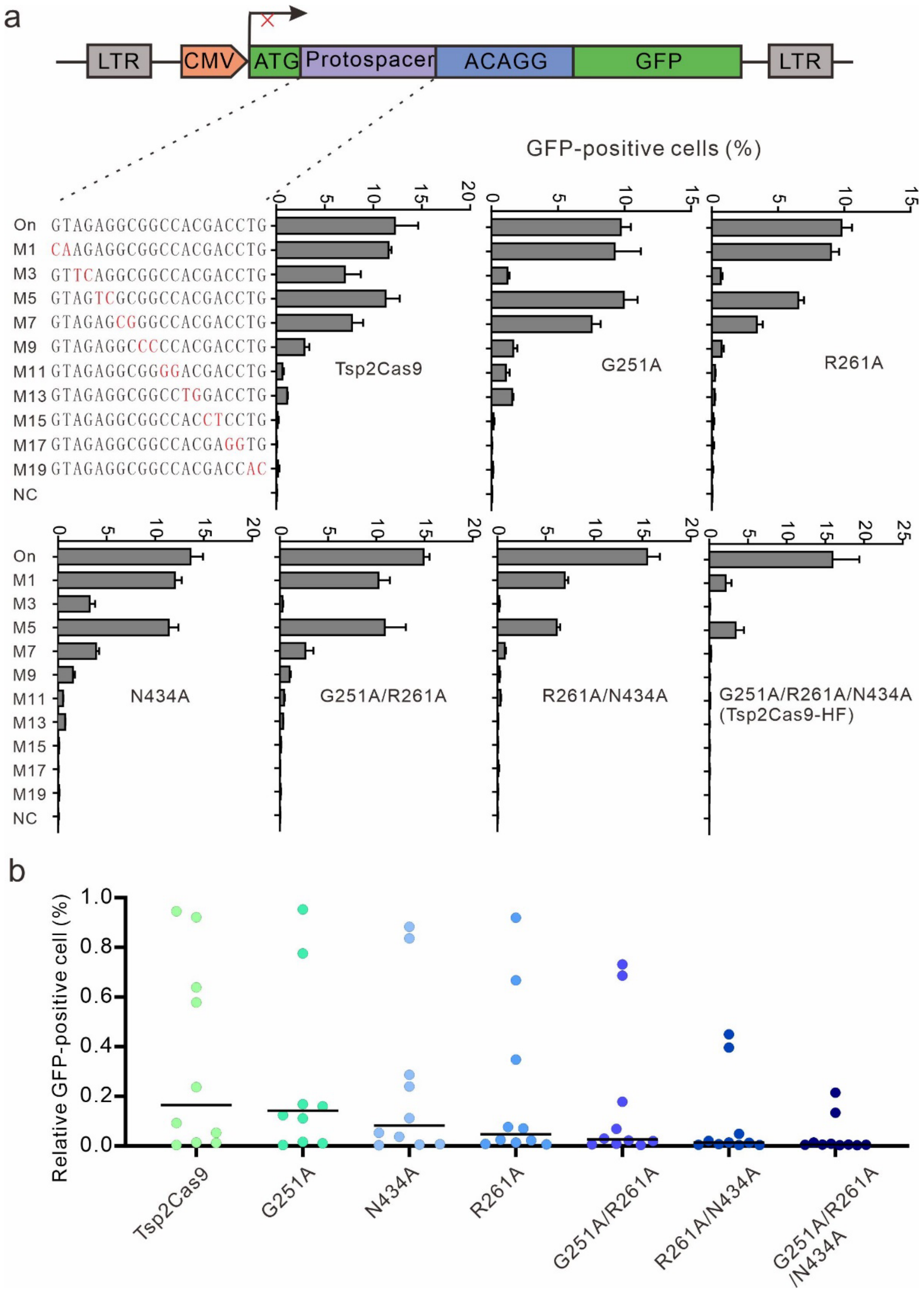

Supplementary Fig. S7 Analysis of mutations on Tsp2Cas9 specificity with the GFP-activation assay. (A) A panel of ten sgRNAs with dinucleotide mutations is used to evaluate specificity. A Schematic of the GFP-activation assay is shown above. sgRNAs and their activity are shown below (n=3). (B) Comparison of editing efficiency at off-targets based on GFP-activation assay among wild-type Tsp2Cas9 and six variants in HEK293T cells. The editing efficiency at off-targets is normalized by on-target efficiency.

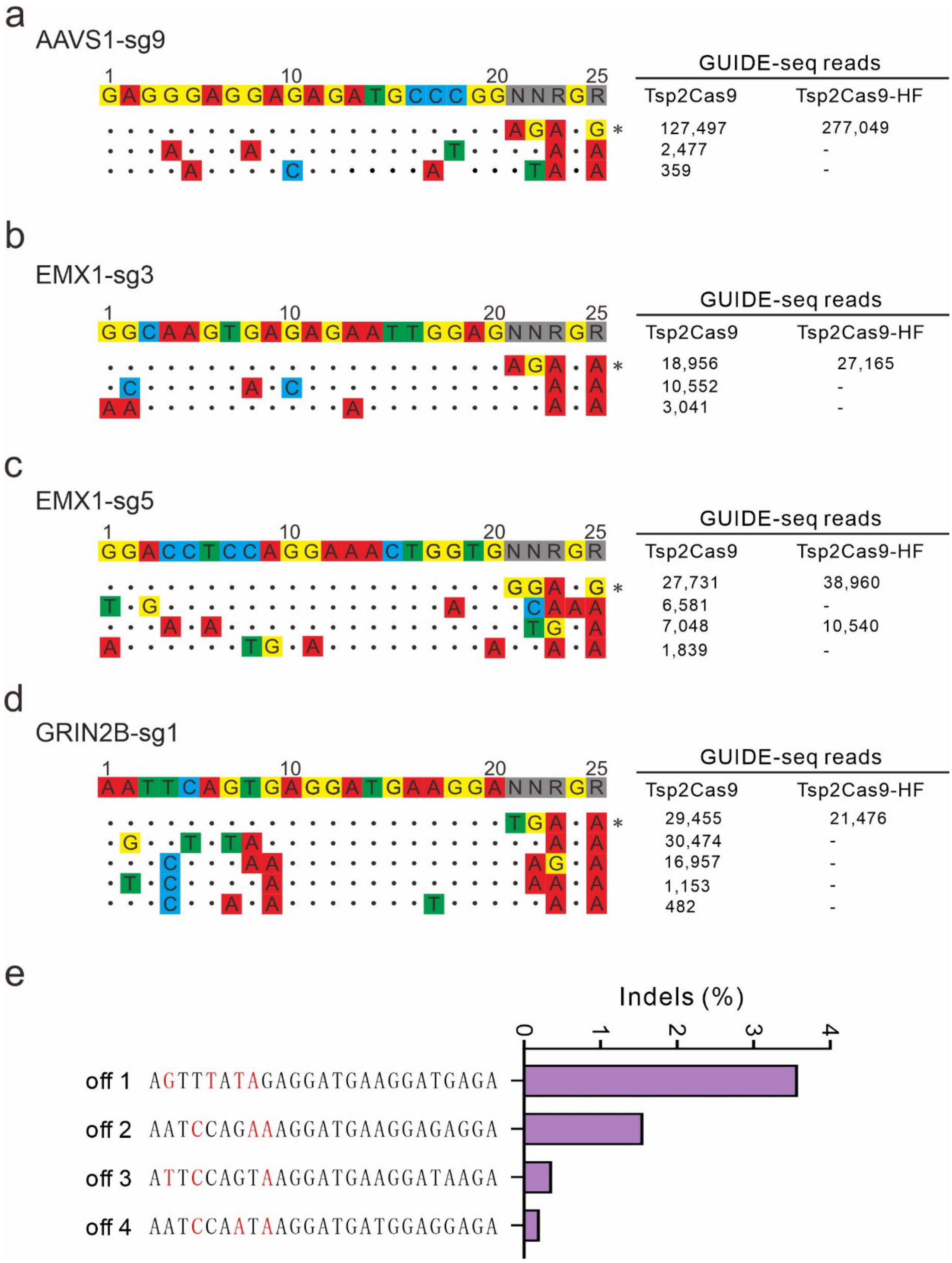

Supplementary Fig. S8 Analysis of Tsp2Cas9 and Tsp2Cas9-HF specificity by GUIDE-seq. Genome-wide analysis of Cas9 specificity by GUIDE-seq at targets AAVS1-sg9 (A), EMX1-sg3 (B), EMX1-sg5 (C), and GRIN2B-sg1 (D). Mismatches compared with the on-target site are shown and highlighted in colour. Read numbers for on- and off-targets are shown on

the right. (E) Off-target cleavage of Tsp2Cas9 for the GRIN2B-sg1 locus is confirmed by targeted deep sequencing.

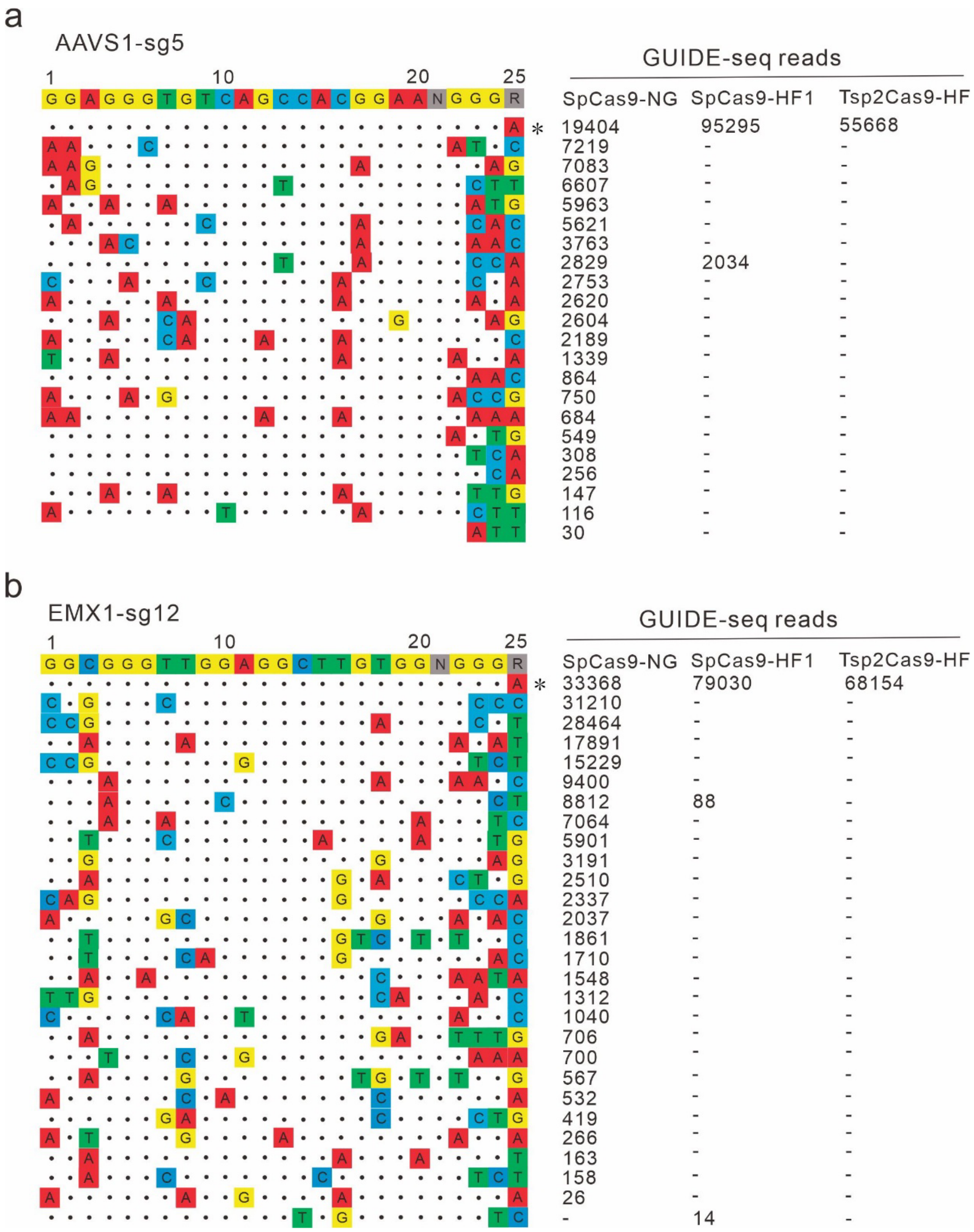

Supplementary Fig. S9 Analysis of SpCas9-NG, SpCas9-HF1, and Tsp2Cas9-HF specificity by GUIDE-seq. Genome-wide analysis of Cas9 specificity by GUIDE-seq at targets AAVS1-sg5 (A), EMX1-sg12 (B), and

GRIN2B-sg5. Mismatches compared with the on-target site are shown and highlighted in colour. Read numbers for on- and off-targets are shown on the right.

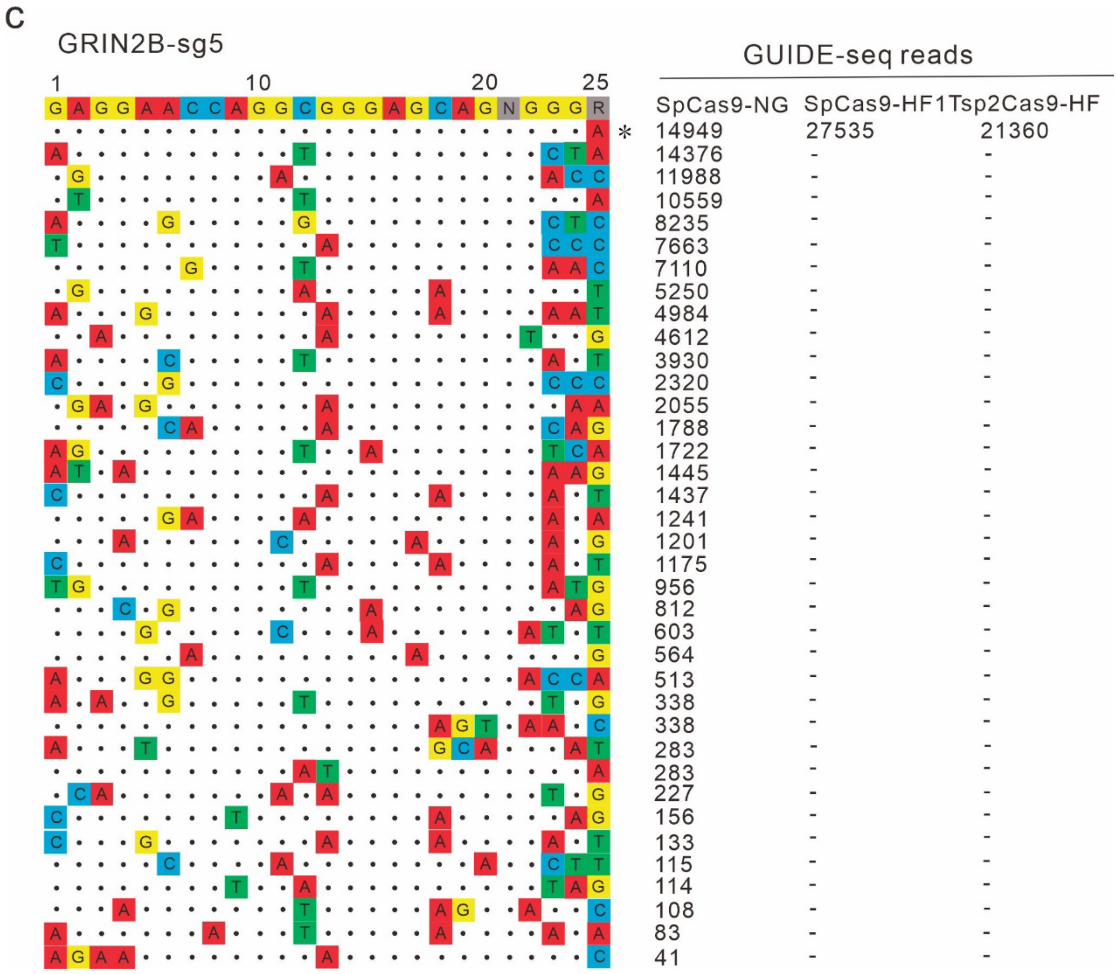

Supplementary Fig. S9 continued.

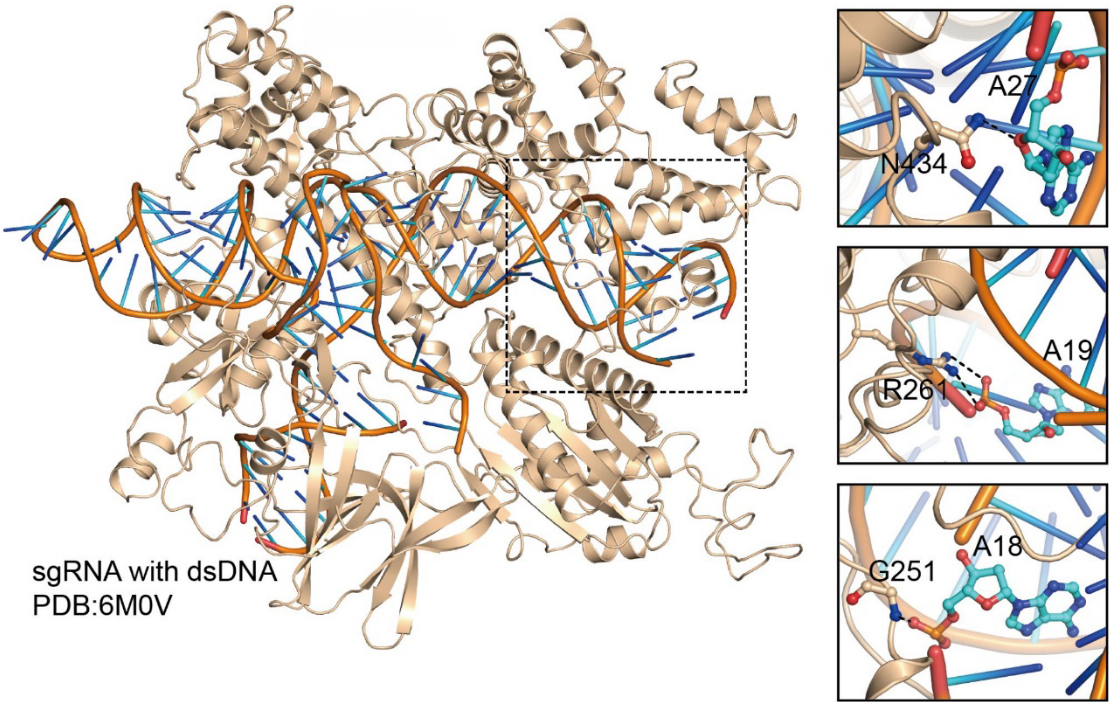

Supplementary Fig. S10 Cartoon representation of SWISS-MODEL predicted structure of Tsp2Cas9 with sgRNA and dsDNA (PDB:6M0V). The Tsp2Cas9 is coloured wheat, and sgRNA and dsDNA are coloured cyan with orange, respectively. Residues involved in the interaction with dsDNA are shown as stick-and-ball models. The dotted lines represent possible hydrogen bonds. The side chain of N434 formed a hydrogen bond with the oxygen atom of the deoxyribose sugar, while the side chain of R261 formed a hydrogen bond with the oxygen atom of the phosphoric acid. Mutating these residues to alanine abolishes their ability to engage in hydrogen bonding interactions. The interaction between G251 and substrate dsDNA is mediated by a hydrogen bond involving the main-chain nitrogen atom of G251. Though this main-chain nitrogen is kept intact upon the mutation of G251 to alanine, considering the unique steric property of the glycine residue, G251A mutation may cause a local conformational alteration, thereby abolishing the hydrogen bond and the interaction between Tsp2Cas9 and dsDNA.

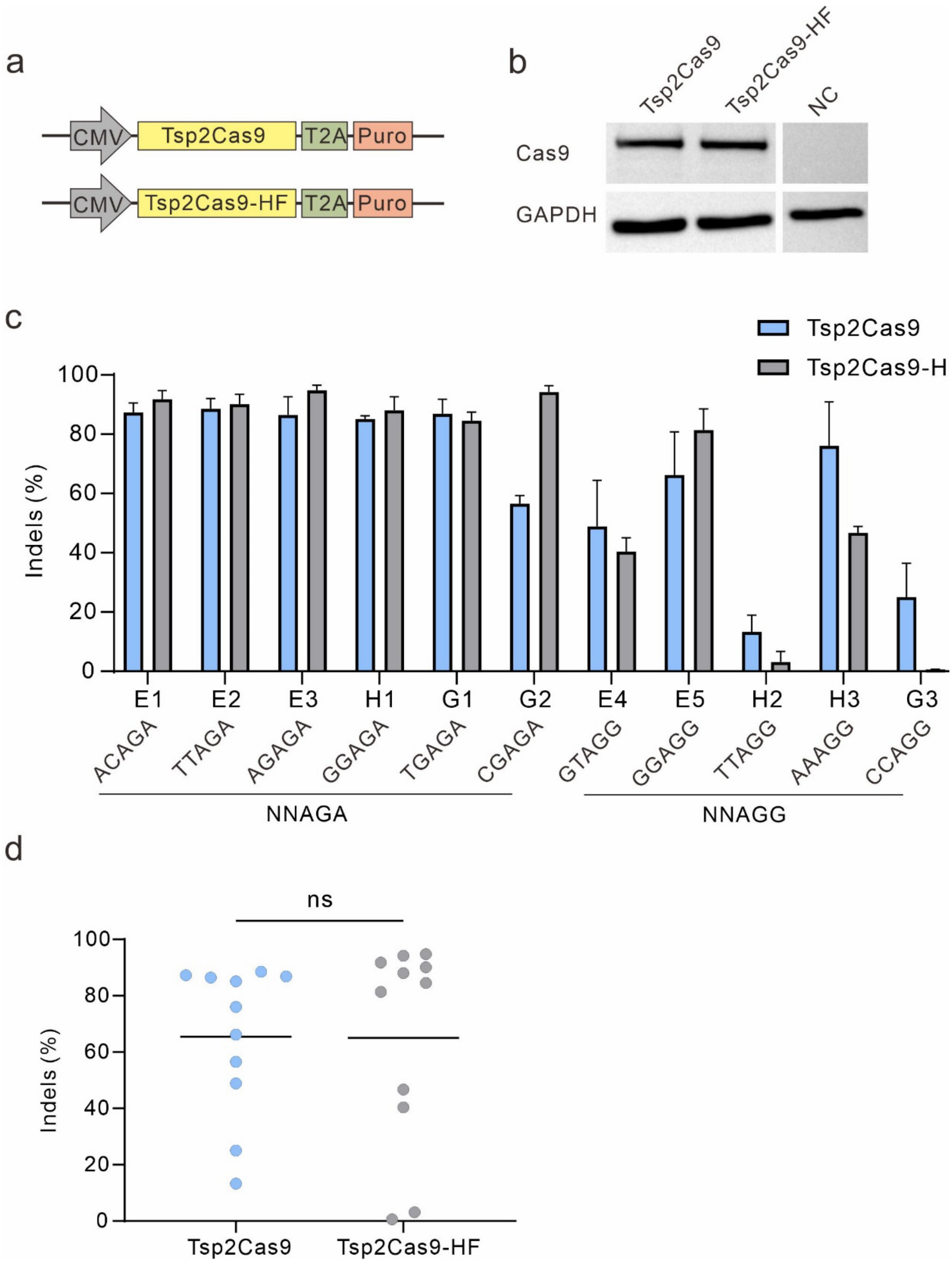

Supplementary Fig. S11 Comparison of wild-type Tsp2Cas9 and Tsp2Cas9-HF activity in HEK293T cells. (A) Schematic of Cas9 expression constructs. (B) Cas9 expression levels are detected by western blot. GAPDH is used as a control. NC: cells without Cas9 transfection are used as a negative control. (C) Comparison of Tsp2Cas9-HF and wild-type Tsp2Cas9

activity at a panel of ten endogenous loci (n=3). (D) Quantification of Tsp2Cas9 and Tsp2Cas9-HF activity.

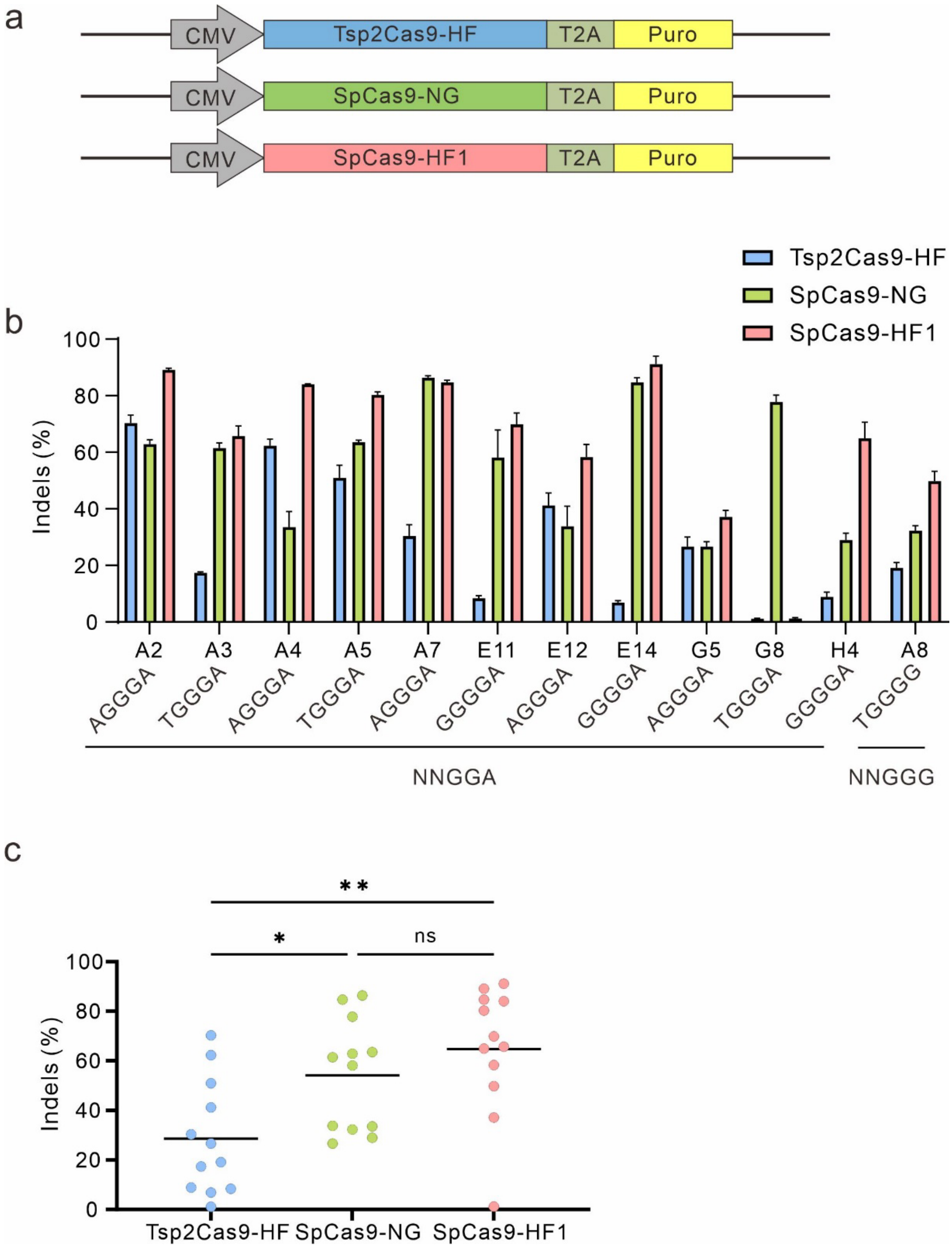

Supplementary Fig. S12 Comparison of Tsp2Cas9-HF, SpCas9-NG, and SpCas9-HF1 activity in HEK293T cells. (A) Schematic of Cas9 expression constructs. (B) Comparison of sp2Cas9-HF, SpCas9-NG, and SpCas9-HF1

activity at a panel of 12 endogenous loci (n=3). (C) Quantification of indel efficiency. A value of P < 0.05 was considered to be statistically significant (*P < 0.05, **P < 0.01, ***P < 0.001, **** P < 0.0001).

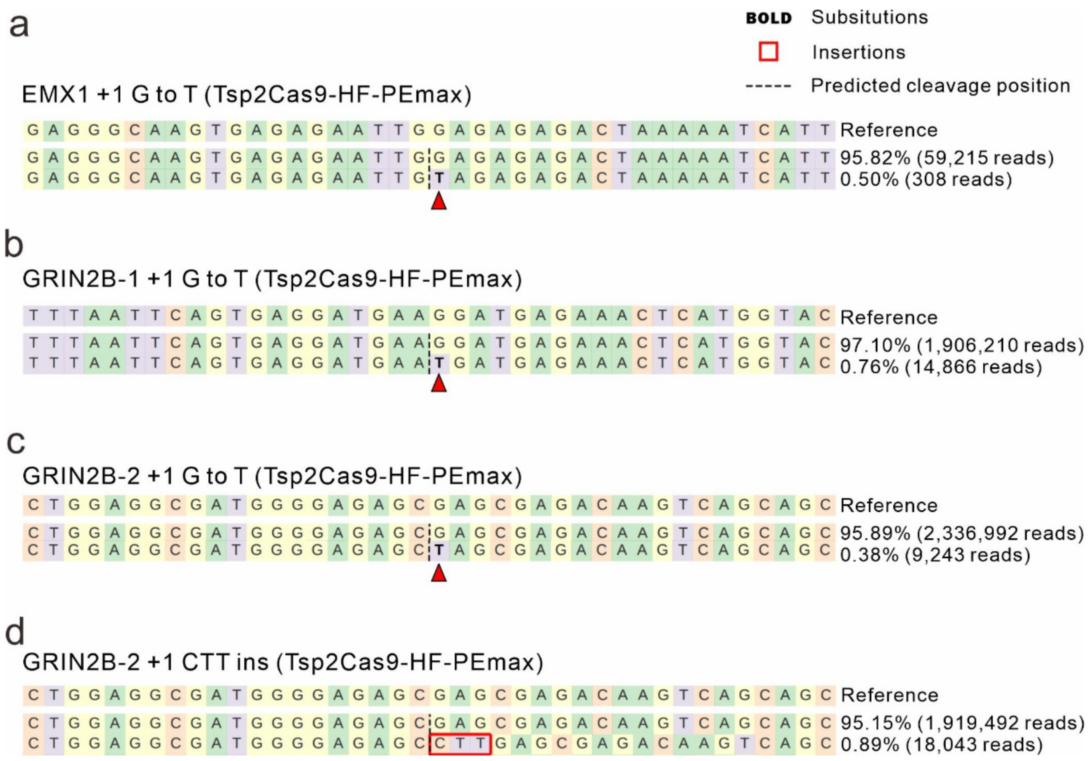

Supplementary Fig. S13 Prime editing with Tsp2Cas9-HF-PEmax induced desired sequences in HEK293T cells. (A-D) Examples of correctly edited reads after prime editing. Nucleotides are indicated by unique colours (A = green; C = red; G = yellow; T = purple). Substitutions are shown in bold font and indicated by red triangles. Red rectangles highlight inserted sequences. The vertical dashed line indicates the predicted cleavage site. The percentage of reads is shown on the right.

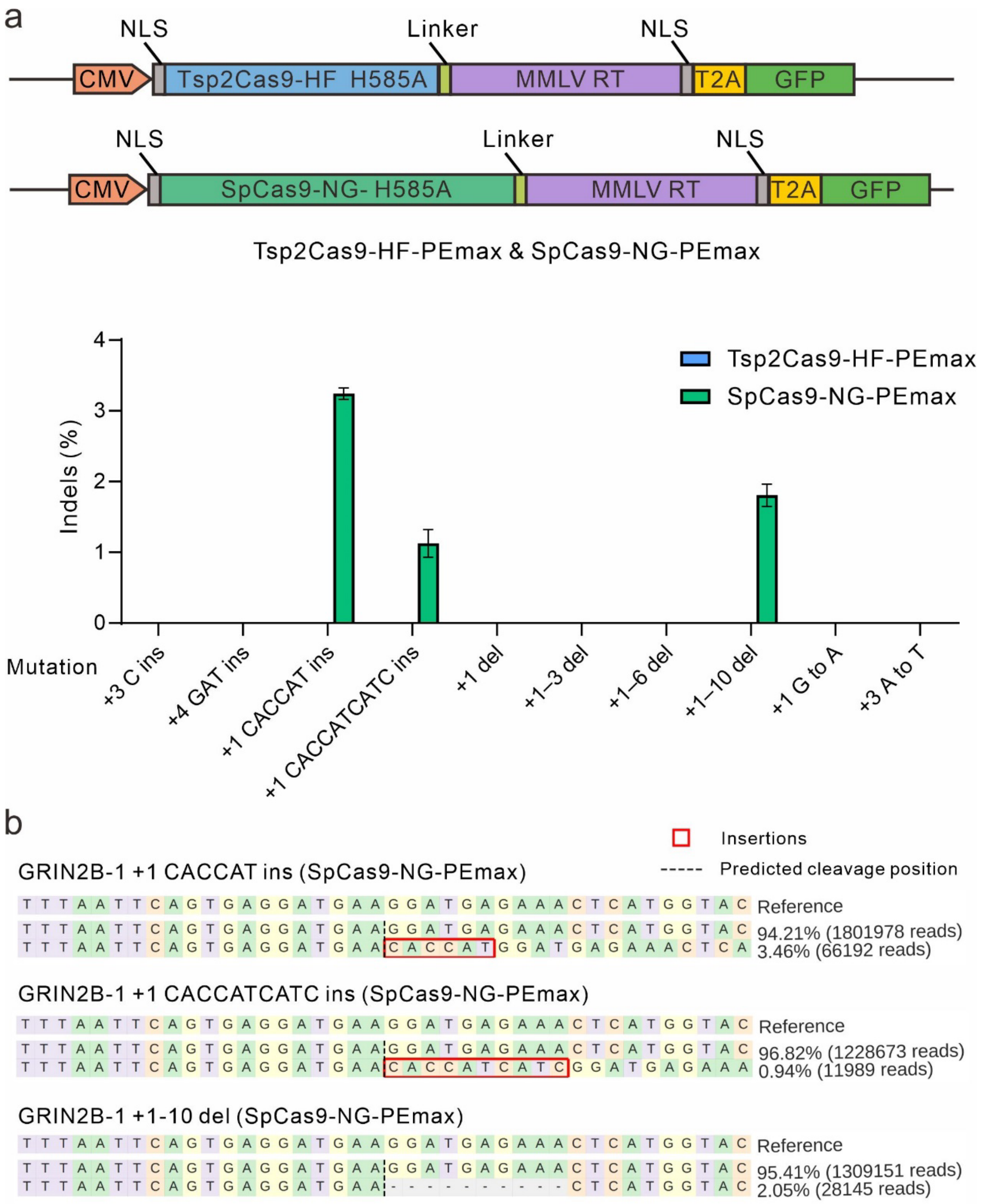

Supplementary Fig. S14 Prime editing with Tsp2Cas9-HF-PEmax and SpCas9-NG-PEmax in HEK293T cells. (A) Prime editing for ten types of mutations at the GRIN2B-1 locus. A schematic of prime editor expression constructs is shown above. PAM and mutation types are shown in the chart below. (B) Examples of correctly edited reads after editing. Nucleotides are indicated by unique colours (A = green; C = red; G = yellow; T = purple). Red rectangles highlight inserted sequences. The vertical dashed line indicates the

predicted cleavage site. The percentage of reads is shown on the right.

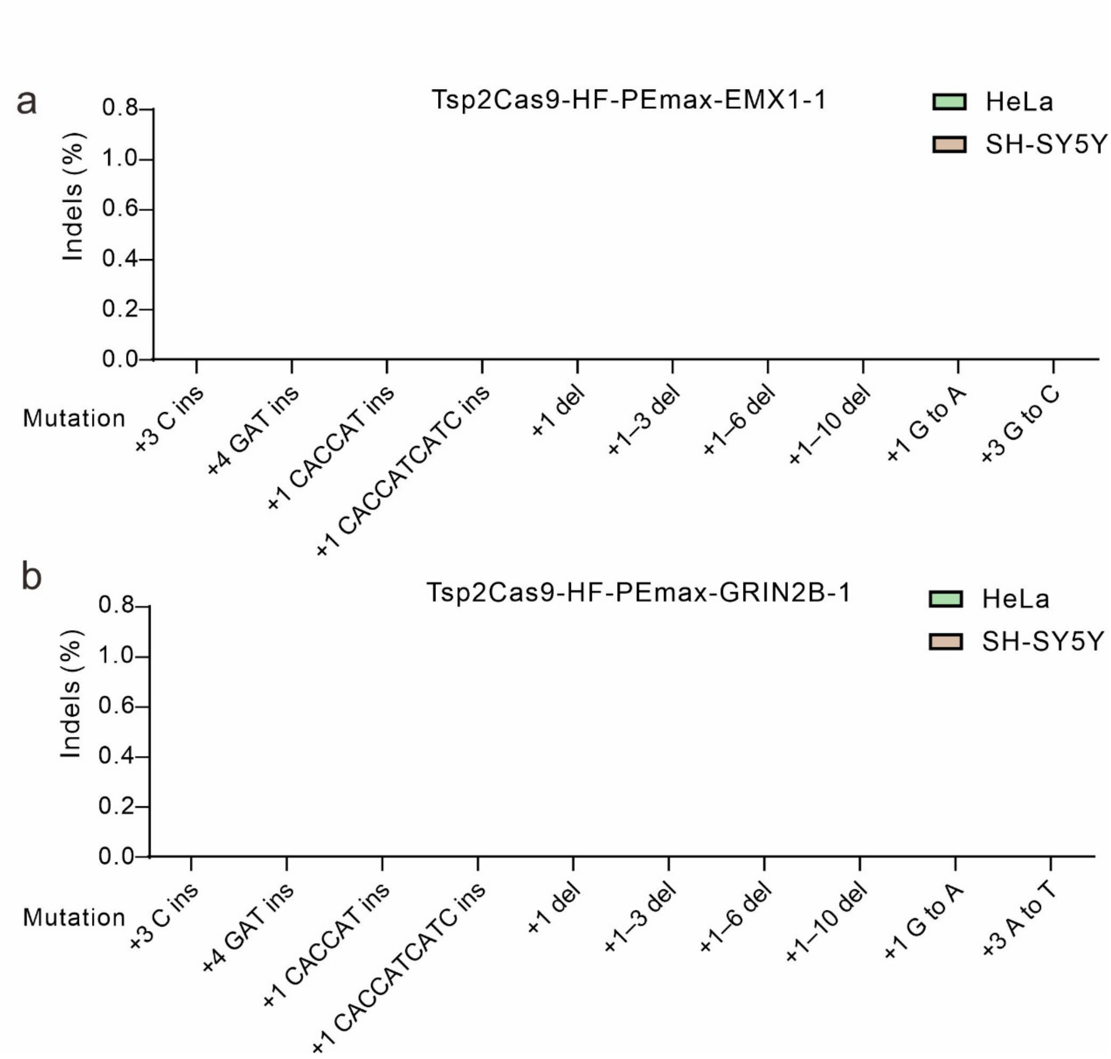

Supplementary Fig. S15 Prime editing of Tsp2Cas9-HF-PEmax in HeLa and SH-SY5Y cells. (A) Prime editing of Tsp2Cas9-HF-PEmax at the EMX1-1 locus. (B) Prime editing of Tsp2Cas9-HF-PEmax at the GRIN2B-1 locus. Mutation types are shown below the chart.

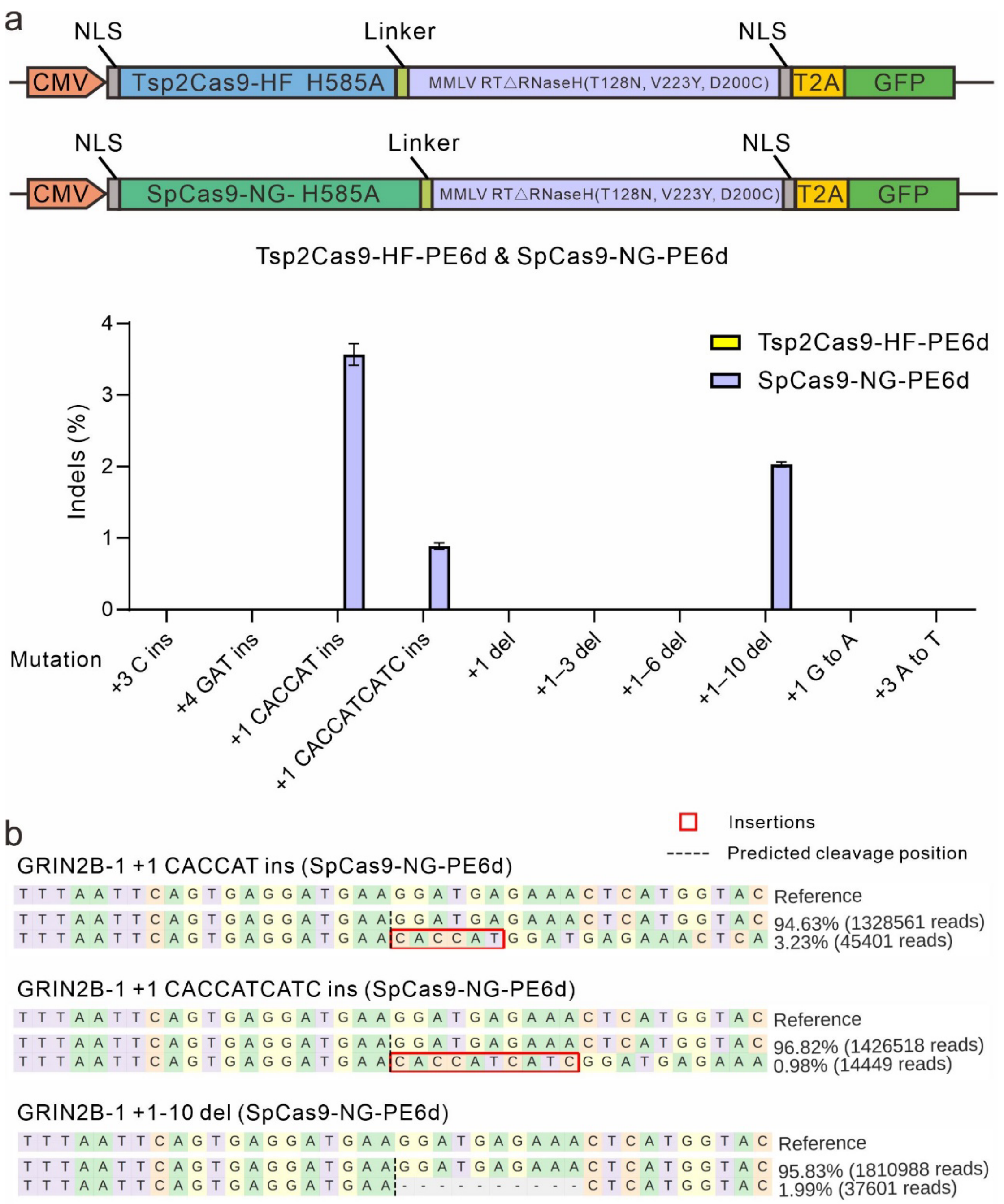

Supplementary Fig. S16 Prime editing with Tsp2Cas9-HF-PE6d and SpCas9-NG-PE6d in HEK293T cells. (A) Prime editing for ten types of mutations at the GRIN2B-1 locus. A schematic of prime editor expression constructs is shown above. PAM and mutation types are shown in the chart below. (B) Examples of correctly edited reads after editing. Nucleotides are indicated by unique colours (A = green; C = red; G = yellow; T = purple). Red rectangles highlight inserted sequences. The vertical dashed line indicates the predicted cleavage site. The percentage of reads is shown on the right.

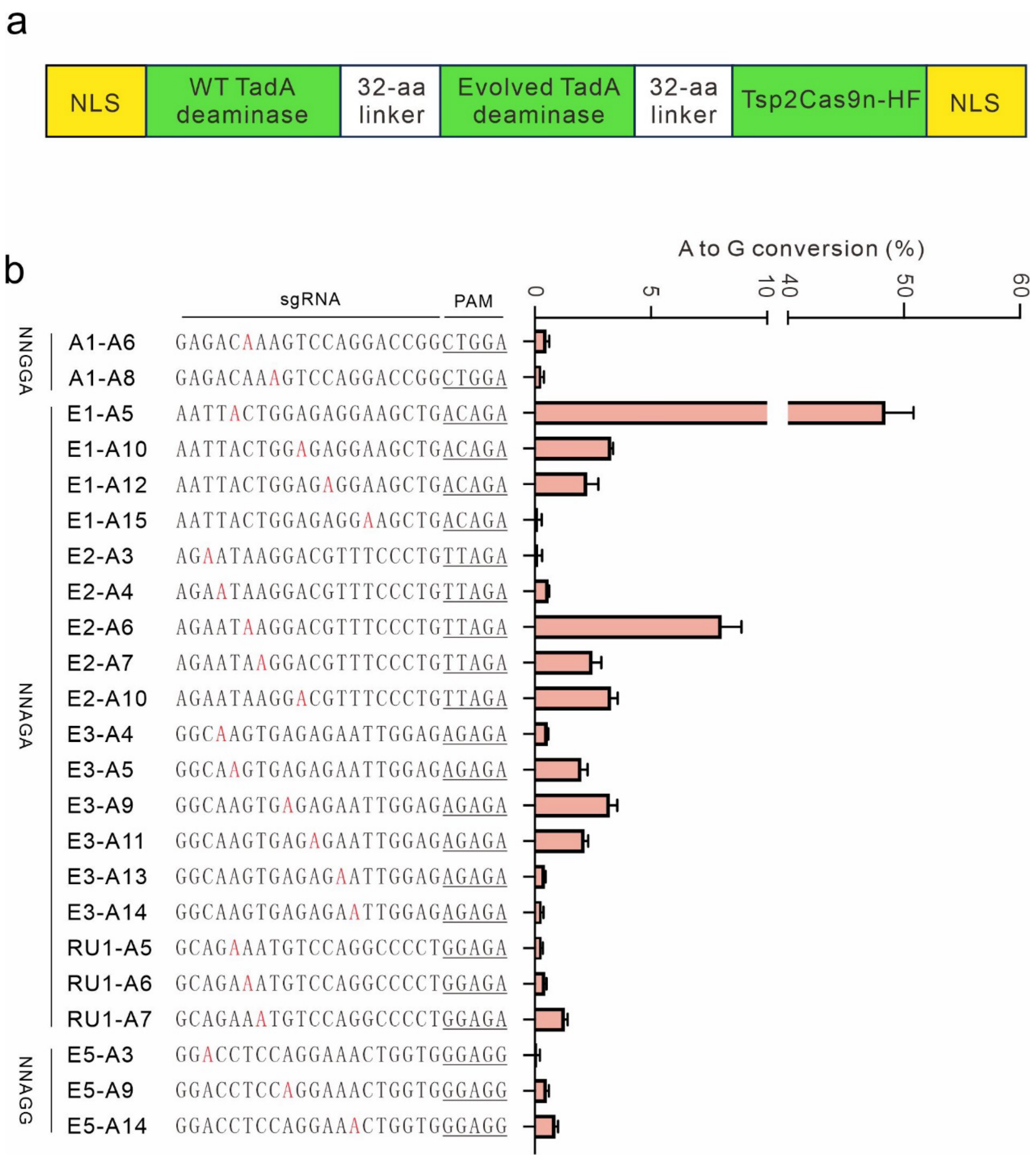

Supplementary Fig. S17 Base editing with Tsp2Cas9-HF-ABEmax.

(A) Schematic representation of Tsp2Cas9-HF-ABEmax structure. (B) Tsp2Cas9-HF-ABEmax enables genome editing at a panel of six endogenous loci (n=3). The target sites and sequences are depicted on the left, with edited adenines highlighted in red and PAMs underlined. 'A1-A6' signifies A at the target A1 position 6.

Sequences S1. Sequences of Tsp2Cas9-HF-PEmax-T2A-GFP

NLS-Tsp2Cas9-HF(H585A)-Linker-MMLV RT(condon opt.)-NLS-T2A-GFP

CCAAAGAAGAAGCGGAAAGTCGCCATGGGCCTGTCTCTGGGCCTGGAC
CTGGGGATTGGCAGCGTCGGATACGGGCTGATTGACATCGAAGATGGCA
CAATCCTGGAGGCCGGCGTGCGGCTGTTTTCCAGCGTAAACCCCGAAAA
CAACCAAACAAGACGGGGATCCAGACAAGCTAGACGGCTGAACAGAAGA
AAAGTGCACAGACTGACCAGAATCGCCCACCTCCTGGAAGAAAGCGGCT
TTTTCAGAGGCGACGTGATGGCCCTGACACCTTACCACCTGAGAGTGAA
AGGCCTGTCCGAAAAGCTGTCCCGGGAGGAACTGTACGCCGCCCTGTA
CAACCTGGCCAAGCATAGAGGCATCAGCTACCTGGAAGATGTGGAACTG
GACGAAGCCGACGGCTCTAGCCTGAGTCACAACGTGGAGCTGCTGAAA
GAGTACTACCCCTGCCAGATCCAGATGGCCAGATACGAGACCTACGGCC
AGATCAGAGGCATCATCGAAACATTTACCAAGGAGGGCGAACCTGAGCT
GCTGGTGAACATCTTCCCTACAAGCGCCTACGAAAAAGAGGCTAGAGCC
ATCCTGAGCAAGCAGGGCGAGTTCTACCCTGAGATCGATATCAAGTTCGT
GGAAAGCTACATTAAGATCCTGACAGATAAACGGCTGTACTATGTGGGCC
CTGGCAACGAGAAGTCTAGAACAGACTACGGCATCTACAGAGAGAATGG
CGAAACCCTGGACAACCTGTTCGACATCCTAATCGGAAAATGTACCGTGT
ACCCCGACGAGAGAAGGGCGGCTAAATTCAGCTACACAGCCCAGCTGTT
TGATTTCCTAAACGACATGAACAACCTGTCTATCGCCGGCGAGAAGCTGA
CAGAGGACCAGAAGCGAAGCATCTACCAGCAGATCATGAGCAGCAAGAC
CGTGAACATGATGAAAATCATCATGAAGGTGACCGGATGTATCAAGGAGG
ATATCAAGGGCTTCAGAGTGGACAACAAGGACAAGCCTGAATTCCACACC
TTCGAGGCTTACAGAAAGTTCCGGAACAAGATGAAGGAGGTGAACGTGG
ATATCACCACCTGGTCCGACGCCGGTTTCGACGAACTGGCTGTCCAGCT
GACCTTGAGCACTGAGTACAAACAGATCGTGGAGCAGGTGAAGAAGCTG
AACCTGCTGCTGTCCGATGAGGCCATCGACGCCATCGCCGACTTCCGGA

AGAAGAATGGCACCTTGTTCTCTAAATGGCACAGTCTGTCCCTGAAGGCC
ATGCACGAGATCATGGACGACCTGTGGAGCACCCATAAGAACCAGATGC
AGCTGTACACCGAACTGGGCGTCTTTAAGAGCAACAAAGACCTGTACAA
GGGACTGAAATACATCAAAAAGGAGATGCTGCTGGAAGAAATCTACAATC
CTGTGGTGAGAGGCAGCATTAGCGAGTCTATCAAGGTGATCAACATGATG
CTGAAGAAGTACGGCGCCCTGGACAAGATCGTGCTGGAAATGCCTAGAG
ATGCTAATGAGAAAGAAGAAAAGAAGAGACTCGATCAGGCCAAGAAGGAT
AACAAGACCCACAAGGATACAGCCATCAAAGTGGCCCAGGAGGAATACG
GCTTCAGCGAGGAAGCCTATCGGCACCACCAGGACCTGAATACCAAGCT
GAGACTCTGGTACGAGCAGGGCAAGTGCTGTCTGTACAGCGGCAAGAC
CATCAAAGTGATGGATCTGATAATGAACCCAAACCAGTTCGACATCGACG
CCATCATTCCCAAGAGCATCAGCTTCGACGACAGCCTGAACAACAAGGT
GCTCTGCTACGCAAGCGAGAACAGATGGAAGGGCAACCAGACCCCTTTC
CGGTATTTCAAGAGGAAGCAGAACAGCCAGTGGAACTACGACAAGTATAA
GTCCCAGATCGTGAAACTGTACAATGACGGCAAGGGCAAACTGACAAGA
GCCAAGATGGACCTGCTGCTGTTTGAAGAGGACATCAATAAATATGAGGT
TAGACGGCAGTTCATCAACAGGAACCTAGTTGACACCAGATACGCCAGCA
GAGTGGTGCTGAACACCCTGCAAGACTTTATGAGCGTGAACTACCCTAAC
ACCACCGTGGGCGTGGTTAGAGGAAAGTTCACCGCCCAGCTGCGGAAA
CACTGGGGCATCAAGAAGGACCGGGACGAATCTCACAGCCATCACGCCA
TCGACGCCCTGACAGTCGCCTGCGTGCCATACCTGAGCCTGTGGAAGAA
ACAGGACATCTTCAAGGAAGAGGATATTAGAGTGGACGATTACTCTGTGA
CAGTGAATGAAGAAACCGGCGAAATCATCGAGGACAGCTACAATCAGCT
GGCCTACCAGCCCCCCTTCGAGAAGTTCTTCACAGGCCTGAGACGGGTG
TCCCTCGAGTGCAGATACAGCTACAAAGTCGACAGAAAGTTCAACCGGC
AGGTGGCCGATGCCACAATCTACAGCACCCGGCAGGGAGTGGTGAAGG
AAACCAAGGGCGAGAAGAAGTTCGTGCTGGACCCTGATTCCAAAGAACA
CTACGTGGTTGCTAAGATCAAGAACATCTATGATGATGCTGATGCCAAGC
GGTTCATGGACAGATACAAGAAGGACAAGACCCAGTTTCTGATGTACCAC

CACGACCCTCAGACCTTTGCCATCCTGGAGGACGCCATACGGCAATATTC
TAAGGAGAAGAACCCTTTCGCCATGTACCGGGAAGAGCACGGCCCTCTG
CAAAAATACGCCAAGAAGGGCAATGGCCCAGAGATCCGGAGCGTCAAAT
ACTTCGACAAGAAGCTGGGAACCCACATCGAGCTGAAGCAGGAGCATGA
GAAGAACCGCAAGGTGGTGCTGCAGAGCCTGAACCCTTGGCGGAGCGA
CATCTACTACAACAAAAAAGAGAACCGGTACATGGTGGCCGGCATCAAGT
ATGCAGACCTTAGCTATCAAAAGGGCAGCGGAGAATACGGAATTTCTCTG
CAGAAGTACCAGCAAGTGCTGAAGTCAGAAAAGATCCTCCTCTCCCTGG
AGGAACTGGAGGCCATCCTTAATGGCGAGTCTACAGATAGCAATTACGAG
TTCTGCTTCAGCCTGTACAAGAACAACCTGCTGCAGTGGACCGATGAAAA
GGACCAGGTGCACCAGTACCGTTTCCTGTCTAGAAACCTGTCTAACAGCA
ATATGATCGAGGTGAAGCCCATCGAGAAATCTAAGTACGAAAAGCAGGCT
TTGGGCAGAAAGACACTGACCAAGGGCATCCACGATTTTTATAAAATCGA
GGTCGACGTGCTGGGCTACCAATACTTCATTAAGCGGGAAAAGCTGAAG
CTGTCATTCAAGTCTAGCTGCTCCGGCGGAAGCTCTGGTGGCAGCAAGC
GGACCGCCGACGGCTCTGAATTCGAGAGCCCTAAGAAGAAAAGAAAGGT
GAGCGGAGGCTCTAGCGGCGGAAGCAGCACCCTGAACATTGAAGACGA
GTATAGACTGCATGAAACAAGCAAGGAACCCGACGTGTCCCTGGGCTCC
ACCTGGCTGTCCGACTTTCCCCAGGCCTGGGCCGAGACAGGAGGAATG
GGCCTGGCCGTGCGGCAGGCACCCCTGATCATCCCTCTGAAGGCCACC
TCTACACCCGTGAGCATCAAGCAGTACCCTATGTCTCAGGAGGCCAGACT
GGGCATCAAGCCTCACATCCAGAGGCTGCTGGACCAGGGCATCCTGGT
GCCATGCCAGAGCCCCTGGAACACACCACTGCTGCCCGTGAAGAAGCC
AGGCACCAATGACTATAGACCCGTGCAGGATCTGAGAGAGGTGAACAAG
AGGGTGGAGGATATCCACCCCACCGTGCCCAACCCTTACAATCTGCTGT
CCGGCCTGCCCCCTTCTCACCAGTGGTATACAGTGCTGGACCTGAAGGA
TGCCTTCTTTTGTCTGAGACTGCACCCTACCAGCCAGCCACTGTTCGCCT
TTGAGTGGAGGGACCCTGAGATGGGCATCTCTGGCCAGCTGACCTGGA
CACGCCTGCCTCAGGGCTTCAAGAATAGCCCAACACTGTTTAACGAGGC

CCTGCACCGCGACCTGGCAGATTTCCGGATCCAGCACCCAGATCTGATC
CTGCTGCAGTACGTGGACGATCTGCTGCTGGCCGCCACCAGCGAGCTG
GATTGCCAGCAGGGAACACGCGCCCTGCTGCAGACCCTGGGAAACCTG
GGATATAGGGCATCCGCCAAGAAGGCCCAGATCTGTCAGAAGCAGGTGA
AGTACCTGGGCTATCTGCTGAAGGAGGGCCAGAGATGGCTGACAGAGGC
CAGGAAGGAGACAGTGATGGGCCAGCCAACACCCAAGACCCCAAGACA
GCTGAGGGAGTTCCTGGGCAAAGCAGGATTTTGCAGGCTGTTCATCCCA
GGATTCGCAGAGATGGCAGCACCTCTGTACCCACTGACCAAGCCGGGCA
CCCTGTTTAATTGGGGCCCTGACCAGCAGAAGGCCTATCAGGAGATCAA
GCAGGCCCTGCTGACAGCACCAGCCCTGGGCCTGCCAGACCTGACCAA
GCCTTTCGAGCTGTTTGTGGATGAGAAGCAGGGCTACGCCAAGGGCGTG
CTGACCCAGAAGCTGGGACCATGGAGACGGCCCGTGGCCTATCTGTCCA
AGAAGCTGGACCCAGTGGCAGCAGGATGGCCACCATGCCTGAGGATGG
TGGCAGCAATCGCCGTGCTGACAAAGGATGCCGGCAAGCTGACCATGG
GACAGCCACTGGTCATCCTGGCACCACACGCAGTGGAGGCCCTGGTGA
AGCAGCCTCCAGATCGCTGGCTGTCTAACGCCCGGATGACACACTACCA
GGCCCTGCTGCTGGACACCGATCGCGTGCAGTTTGGCCCTGTGGTGGC
CCTGAATCCAGCCACCCTGCTGCCTCTGCCAGAGGAGGGCCTGCAGCA
CAACTGTCTGGACATCCTGGCAGAGGCACACGGAACAAGGCCAGACCT
GACCGATCAGCCCCTGCCTGACGCCGATCACACATGGTATACCGATGGA
AGCTCCCTGCTGCAGGAGGGCCAGAGGAAGGCAGGAGCAGCAGTGAC
CACAGAGACAGAAGTGATCTGGGCCAAGGCCCTGCCAGCAGGCACATC
CGCCCAGCGGGCCGAGCTGATCGCCCTGACCCAGGCCCTGAAGATGGC
CGAGGGCAAGAAGCTGAACGTGTACACAGACTCCAGATATGCCTTCGCC
ACCGCACACATCCACGGAGAGATCTACAGGCGCCGGGGCTGGCTGACC
TCTGAGGGCAAGGAGATCAAGAACAAGGATGAGATCCTGGCCCTGCTGA
AGGCCCTGTTTCTGCCCAAGCGGCTGAGCATCATCCACTGTCCTGGACA
CCAGAAGGGACACTCCGCCGAGGCAAGGGGCAATCGGATGGCCGACCA
GGCCGCCAGAAAGGCTGCTATTACTGAAACTCCCGACACTTCCACTCTG

CTGATTGAAAACTCCTCCCCTTCTGGCGGCTCAAAAAGAACCGCCGACG
GCAGCGAATTCGAGCCCAAGAAGAAGAGGAAAGTCGGCTCTGGCCCTG
CCGCTAAGAGAGTGAAGCTGGACGGATCCGGCGAGGGCAGAGGAAGTC
TGCTAACATGCGGTGACGTCGAGGAGAATCCTGGCCCAGTGAGCAAGG
GCGAGGAGCTGTTCACCGGGGTGGTGCCCATCCTGGTCGAGCTGGACG
GCGACGTAAACGGCCACAAGTTCAGCGTGTCCGGCGAGGGCGAGGGCG
ATGCCACCTACGGCAAGCTGACCCTGAAGTTCATCTGCACCACCGGCAA
GCTGCCCGTGCCCTGGCCCACCCTCGTGACCACCCTGACCTACGGCGT
GCAGTGCTTCAGCCGCTACCCCGACCACATGAAGCAGCACGACTTCTTC
AAGTCCGCCATGCCCGAAGGCTACGTCCAGGAGCGCACCATCTTCTTCA
AGGACGACGGCAACTACAAGACCCGCGCCGAGGTGAAGTTCGAGGGCG
ACACCCTGGTGAACCGCATCGAGCTGAAGGGCATCGACTTCAAGGAGGA
CGGCAACATCCTGGGGCACAAGCTGGAGTACAACTACAACAGCCACAAC
GTCTATATCATGGCCGACAAGCAGAAGAACGGCATCAAGGTGAACTTCAA
GATCCGCCACAACATCGAGGACGGCAGCGTGCAGCTCGCCGACCACTA
CCAGCAGAACACCCCCATCGGCGACGGCCCCGTGCTGCTGCCCGACAA
CCACTACCTGAGCACCCAGTCCGCCCTGAGCAAAGACCCCAACGAGAAG
CGCGATCACATGGTCCTGCTGGAGTTCGTGACCGCCGCCGGGATCACTC
TCGGCATGGACGAGCTGTACAAGTAA

Sequences S2. Sequences of SpCas9-NG-PEmax-T2A-GFP

NLS-SpCas9-NG(H840A)-Linker-MMLV RT(condon opt.)-NLS-T2A-GFP

CCAAAGAAGAAGCGGAAAGTCGACAAGAAGTACAGCATCGGCCTGGACA
TCGGCACCAACTCTGTGGGCTGGGCCGTGATCACCGACGAGTACAAGGT
GCCCAGCAAGAAATTCAAGGTGCTGGGCAACACCGACCGGCACAGCATC
AAGAAGAACCTGATCGGAGCCCTGCTGTTCGACAGCGGCGAAACAGCC
GAGGCCACCCGGCTGAAGAGAACCGCCAGAAGAAGATACACCAGACGG

AAGAACCGGATCTGCTATCTGCAAGAGATCTTCAGCAACGAGATGGCCAA

GGTGGACGACAGCTTCTTCCACAGACTGGAAGAGTCCTTCCTGGTGGAA

GAGGATAAGAAGCACGAGCGGCACCCCATCTTCGGCAACATCGTGGACG

AGGTGGCCTACCACGAGAAGTACCCCACCATCTACCACCTGAGAAAGAA

ACTGGTGGACAGCACCGACAAGGCCGACCTGCGGCTGATCTATCTGGCC

CTGGCCCACATGATCAAGTTCCGGGGCCACTTCCTGATCGAGGGCGACC

TGAACCCCGACAACAGCGACGTGGACAAGCTGTTCATCCAGCTGGTGCA

GACCTACAACCAGCTGTTCGAGGAAAACCCCATCAACGCCAGCGGCGTG

GACGCCAAGGCCATCCTGTCTGCCAGACTGAGCAAGAGCAGACGGCTG

GAAAATCTGATCGCCCAGCTGCCCGGCGAGAAGAAGAATGGCCTGTTCG

GAAACCTGATTGCCCTGAGCCTGGGCCTGACCCCCAACTTCAAGAGCAA

CTTCGACCTGGCCGAGGATGCCAAACTGCAGCTGAGCAAGGACACCTAC

GACGACGACCTGGACAACCTGCTGGCCCAGATCGGCGACCAGTACGCC

GACCTGTTTCTGGCCGCCAAGAACCTGTCCGACGCCATCCTGCTGAGCG

ACATCCTGAGAGTGAACACCGAGATCACCAAGGCCCCCCTGAGCGCCTC

TATGATCAAGAGATACGACGAGCACCACCAGGACCTGACCCTGCTGAAA

GCTCTCGTGCGGCAGCAGCTGCCTGAGAAGTACAAAGAGATTTTCTTCG

ACCAGAGCAAGAACGGCTACGCCGGCTACATTGACGGCGGAGCCAGCC

AGGAAGAGTTCTACAAGTTCATCAAGCCCATCCTGGAAAAGATGGACGGC

ACCGAGGAACTGCTCGTGAAGCTGAACAGAGAGGACCTGCTGCGGAAG

CAGCGGACCTTCGACAACGGCAGCATCCCCCACCAGATCCACCTGGGA

GAGCTGCACGCCATTCTGCGGCGGCAGGAAGATTTTTACCCATTCCTGAA

GGACAACCGGGAAAAGATCGAGAAGATCCTGACCTTCCGCATCCCCTAC

TACGTGGGCCCTCTGGCCAGGGGAAACAGCAGATTCGCCTGGATGACCA

GAAAGAGCGAGGAAACCATCACCCCCTGGAACTTCGAGGAAGTGGTGG

ACAAGGGCGCTTCCGCCCAGAGCTTCATCGAGCGGATGACCAACTTCGA

TAAGAACCTGCCCAACGAGAAGGTGCTGCCCAAGCACAGCCTGCTGTAC

GAGTACTTCACCGTGTATAACGAGCTGACCAAAGTGAAATACGTGACCGA

GGGAATGAGAAAGCCCGCCTTCCTGAGCGGCGAGCAGAAAAAGGCCAT

CGTGGACCTGCTGTTCAAGACCAACCGGAAAGTGACCGTGAAGCAGCT
GAAAGAGGACTACTTCAAGAAAATCGAGTGCTTCGACTCCGTGGAAATCT
CCGGCGTGGAAGATCGGTTCAACGCCTCCCTGGGCACATACCACGATCT
GCTGAAAATTATCAAGGACAAGGACTTCCTGGACAATGAGGAAAACGAGG
ACATTCTGGAAGATATCGTGCTGACCCTGACACTGTTTGAGGACAGAGAG
ATGATCGAGGAACGGCTGAAAACCTATGCCCACCTGTTCGACGACAAAGT
GATGAAGCAGCTGAAGCGGCGGAGATACACCGGCTGGGGCAGGCTGAG
CCGGAAGCTGATCAACGGCATCCGGGACAAGCAGTCCGGCAAGACAATC
CTGGATTTCCTGAAGTCCGACGGCTTCGCCAACAGAAACTTCATGCAGCT
GATCCACGACGACAGCCTGACCTTTAAAGAGGACATCCAGAAAGCCCAG
GTGTCCGGCCAGGGCGATAGCCTGCACGAGCACATTGCCAATCTGGCCG
GCAGCCCCGCCATTAAGAAGGGCATCCTGCAGACAGTGAAGGTGGTGG
ACGAGCTCGTGAAAGTGATGGGCCGGCACAAGCCCGAGAACATCGTGAT
CGAAATGGCCAGAGAGAACCAGACCACCCAGAAGGGACAGAAGAACAG
CCGCGAGAGAATGAAGCGGATCGAAGAGGGCATCAAAGAGCTGGGCAG
CCAGATCCTGAAAGAACACCCCGTGGAAAACACCCAGCTGCAGAACGAG
AAGCTGTACCTGTACTACCTGCAGAATGGGCGGGATATGTACGTGGACCA
GGAACTGGACATCAACCGGCTGTCCGACTACGATGTGGACGCCATCGTG
CCTCAGAGCTTTCTGAAGGACGACTCCATCGACAACAAGGTGCTGACCA
GAAGCGACAAGAACCGGGGCAAGAGCGACAACGTGCCCTCCGAAGAGG
TCGTGAAGAAGATGAAGAACTACTGGCGGCAGCTGCTGAACGCCAAGCT
GATTACCCAGAGAAAGTTCGACAATCTGACCAAGGCCGAGAGAGGCGGC
CTGAGCGAACTGGATAAGGCCGGCTTCATCAAGAGACAGCTGGTGGAAA
CCCGGCAGATCACAAAGCACGTGGCACAGATCCTGGACTCCCGGATGAA
CACTAAGTACGACGAGAATGACAAGCTGATCCGGGAAGTGAAAGTGATCA
CCCTGAAGTCCAAGCTGGTGTCCGATTTCCGGAAGGATTTCCAGTTTTAC
AAAGTGCGCGAGATCAACAACTACCACCACGCCCACGACGCCTACCTGA
ACGCCGTCGTGGGAACCGCCCTGATCAAAAAGTACCCTAAGCTGGAAAG
CGAGTTCGTGTACGGCGACTACAAGGTGTACGACGTGCGGAAGATGATC

GCCAAGAGCGAGCAGGAAATCGGCAAGGCTACCGCCAAGTACTTCTTCT
ACAGCAACATCATGAACTTTTTCAAGACCGAGATTACCCTGGCCAACGGC
GAGATCCGGAAGCGGCCTCTGATCGAGACAAACGGCGAAACCGGGGAG
ATCGTGTGGGATAAGGGCCGGGATTTTGCCACCGTGCGGAAAGTGCTGA
GCATGCCCCAAGTGAATATCGTGAAAAAGACCGAGGTGCAGACAGGCGG
CTTCAGCAAAGAGTCTATCAGGCCCAAGAGGAACAGCGATAAGCTGATC
GCCAGAAAGAAGGACTGGGACCCTAAGAAGTACGGCGGCTTCGTCAGC
CCCACCGTGGCCTATTCTGTGCTGGTGGTGGCCAAAGTGGAAAAGGGCA
AGTCCAAGAAACTGAAGAGTGTGAAAGAGCTGCTGGGGATCACCATCAT
GGAAAGAAGCAGCTTCGAGAAGAATCCCATCGACTTTCTGGAAGCCAAG
GGCTACAAAGAAGTGAAAAAGGACCTGATCATCAAGCTGCCTAAGTACTC
CCTGTTCGAGCTGGAAAACGGCCGGAAGAGAATGCTGGCCTCTGCCCG
CTTCCTGCAGAAGGGAAACGAACTGGCCCTGCCCTCCAAATATGTGAAC
TTCCTGTACCTGGCCAGCCACTATGAGAAGCTGAAGGGCTCCCCCGAGG
ATAATGAGCAGAAACAGCTGTTTGTGGAACAGCACAAGCACTACCTGGAC
GAGATCATCGAGCAGATCAGCGAGTTCTCCAAGAGAGTGATCCTGGCCG
ACGCTAATCTGGACAAAGTGCTGTCCGCCTACAACAAGCACCGGGATAA
GCCCATCAGAGAGCAGGCCGAGAATATCATCCACCTGTTTACCCTGACCA
ATCTGGGAGCCCCTCGCGCCTTCAAGTACTTTGACACCACCATCGACCG
GAAGGTGTACAGAAGCACCAAAGAGGTGCTGGACGCCACCCTGATCCAC
CAGAGCATCACCGGCCTGTACGAGACACGGATCGACCTGTCTCAGCTGG
GAGGCGACTCCGGCGGAAGCTCTGGTGGCAGCAAGCGGACCGCCGAC
GGCTCTGAATTCGAGAGCCCTAAGAAGAAAAGAAAGGTGAGCGGAGGCT
CTAGCGGCGGAAGCAGCACCCTGAACATTGAAGACGAGTATAGACTGCA
TGAAACAAGCAAGGAACCCGACGTGTCCCTGGGCTCCACCTGGCTGTC
CGACTTTCCCCAGGCCTGGGCCGAGACAGGAGGAATGGGCCTGGCCGT
GCGGCAGGCACCCCTGATCATCCCTCTGAAGGCCACCTCTACACCCGTG
AGCATCAAGCAGTACCCTATGTCTCAGGAGGCCAGACTGGGCATCAAGC
CTCACATCCAGAGGCTGCTGGACCAGGGCATCCTGGTGCCATGCCAGAG

CCCCTGGAACACACCACTGCTGCCCGTGAAGAAGCCAGGCACCAATGAC

TATAGACCCGTGCAGGATCTGAGAGAGGTGAACAAGAGGGTGGAGGATA

TCCACCCCACCGTGCCCAACCCTTACAATCTGCTGTCCGGCCTGCCCCC

TTCTCACCAGTGGTATACAGTGCTGGACCTGAAGGATGCCTTCTTTTGTC

TGAGACTGCACCCTACCAGCCAGCCACTGTTCGCCTTTGAGTGGAGGGA

CCCTGAGATGGGCATCTCTGGCCAGCTGACCTGGACACGCCTGCCTCAG

GGCTTCAAGAATAGCCCAACACTGTTTAACGAGGCCCTGCACCGCGACC

TGGCAGATTTCCGGATCCAGCACCCAGATCTGATCCTGCTGCAGTACGTG

GACGATCTGCTGCTGGCCGCCACCAGCGAGCTGGATTGCCAGCAGGGA

ACACGCGCCCTGCTGCAGACCCTGGGAAACCTGGGATATAGGGCATCCG

CCAAGAAGGCCCAGATCTGTCAGAAGCAGGTGAAGTACCTGGGCTATCT

GCTGAAGGAGGGCCAGAGATGGCTGACAGAGGCCAGGAAGGAGACAGT

GATGGGCCAGCCAACACCCAAGACCCCAAGACAGCTGAGGGAGTTCCT

GGGCAAAGCAGGATTTTGCAGGCTGTTCATCCCAGGATTCGCAGAGATG

GCAGCACCTCTGTACCCACTGACCAAGCCGGGCACCCTGTTTAATTGGG

GCCCTGACCAGCAGAAGGCCTATCAGGAGATCAAGCAGGCCCTGCTGAC

AGCACCAGCCCTGGGCCTGCCAGACCTGACCAAGCCTTTCGAGCTGTTT

GTGGATGAGAAGCAGGGCTACGCCAAGGGCGTGCTGACCCAGAAGCTG

GGACCATGGAGACGGCCCGTGGCCTATCTGTCCAAGAAGCTGGACCCA

GTGGCAGCAGGATGGCCACCATGCCTGAGGATGGTGGCAGCAATCGCC

GTGCTGACAAAGGATGCCGGCAAGCTGACCATGGGACAGCCACTGGTC

ATCCTGGCACCACACGCAGTGGAGGCCCTGGTGAAGCAGCCTCCAGAT

CGCTGGCTGTCTAACGCCCGGATGACACACTACCAGGCCCTGCTGCTGG

ACACCGATCGCGTGCAGTTTGGCCCTGTGGTGGCCCTGAATCCAGCCAC

CCTGCTGCCTCTGCCAGAGGAGGGCCTGCAGCACAACTGTCTGGACATC

CTGGCAGAGGCACACGGAACAAGGCCAGACCTGACCGATCAGCCCCTG

CCTGACGCCGATCACACATGGTATACCGATGGAAGCTCCCTGCTGCAGG

AGGGCCAGAGGAAGGCAGGAGCAGCAGTGACCACAGAGACAGAAGTGA

TCTGGGCCAAGGCCCTGCCAGCAGGCACATCCGCCCAGCGGGCCGAGC

TGATCGCCCTGACCCAGGCCCTGAAGATGGCCGAGGGCAAGAAGCTGA
ACGTGTACACAGACTCCAGATATGCCTTCGCCACCGCACACATCCACGGA
GAGATCTACAGGCGCCGGGGCTGGCTGACCTCTGAGGGCAAGGAGATC
AAGAACAAGGATGAGATCCTGGCCCTGCTGAAGGCCCTGTTTCTGCCCA
AGCGGCTGAGCATCATCCACTGTCCTGGACACCAGAAGGGACACTCCGC
CGAGGCAAGGGGCAATCGGATGGCCGACCAGGCCGCCAGAAAGGCTGC
TATTACTGAAACTCCCGACACTTCCACTCTGCTGATTGAAAACTCCTCCCC
TTCTGGCGGCTCAAAAAGAACCGCCGACGGCAGCGAATTCGAGCCCAA
GAAGAAGAGGAAAGTCGGCTCTGGCCCTGCCGCTAAGAGAGTGAAGCT
GGACGGATCCGGCGAGGGCAGAGGAAGTCTGCTAACATGCGGTGACGT
CGAGGAGAATCCTGGCCCAGTGAGCAAGGGCGAGGAGCTGTTCACCGG
GGTGGTGCCCATCCTGGTCGAGCTGGACGGCGACGTAAACGGCCACAA
GTTCAGCGTGTCCGGCGAGGGCGAGGGCGATGCCACCTACGGCAAGCT
GACCCTGAAGTTCATCTGCACCACCGGCAAGCTGCCCGTGCCCTGGCC
CACCCTCGTGACCACCCTGACCTACGGCGTGCAGTGCTTCAGCCGCTAC
CCCGACCACATGAAGCAGCACGACTTCTTCAAGTCCGCCATGCCCGAAG
GCTACGTCCAGGAGCGCACCATCTTCTTCAAGGACGACGGCAACTACAA
GACCCGCGCCGAGGTGAAGTTCGAGGGCGACACCCTGGTGAACCGCAT
CGAGCTGAAGGGCATCGACTTCAAGGAGGACGGCAACATCCTGGGGCA
CAAGCTGGAGTACAACTACAACAGCCACAACGTCTATATCATGGCCGACA
AGCAGAAGAACGGCATCAAGGTGAACTTCAAGATCCGCCACAACATCGA
GGACGGCAGCGTGCAGCTCGCCGACCACTACCAGCAGAACACCCCCAT
CGGCGACGGCCCCGTGCTGCTGCCCGACAACCACTACCTGAGCACCCA
GTCCGCCCTGAGCAAAGACCCCAACGAGAAGCGCGATCACATGGTCCTG
CTGGAGTTCGTGACCGCCGCCGGGATCACTCTCGGCATGGACGAGCTG
TACAAGTAA

Sequences S3. Sequences of Tsp2Cas9-HF-PE6d-T2A-GFP

NLS-Tsp2Cas9-HF(H585A)-Linker-MMLV RT△RNaseH(T128N, V223Y, D200C)-NLS-T2A-GFP

CCAAAGAAGAAGCGGAAAGTCGCCATGGGCCTGTCTCTGGGCCTGGAC
CTGGGGATTGGCAGCGTCGGATACGGGCTGATTGACATCGAAGATGGC
ACAATCCTGGAGGCCGGCGTGCGGCTGTTTTCCAGCGTAAACCCCGAAA
ACAACCAAACAAGACGGGGATCCAGACAAGCTAGACGGCTGAACAGAA
GAAAAGTGCACAGACTGACCAGAATCGCCCACCTCCTGGAAGAAAGCG
GCTTTTTCAGAGGCGACGTGATGGCCCTGACACCTTACCACCTGAGAGT
GAAAGGCCTGTCCGAAAAGCTGTCCCGGGAGGAACTGTACGCCGCCCT
GTACAACCTGGCCAAGCATAGAGGCATCAGCTACCTGGAAGATGTGGAA
CTGGACGAAGCCGACGGCTCTAGCCTGAGTCACAACGTGGAGCTGCTG
AAAGAGTACTACCCCTGCCAGATCCAGATGGCCAGATACGAGACCTACG
GCCAGATCAGAGGCATCATCGAAACATTTACCAAGGAGGGCGAACCTGA
GCTGCTGGTGAACATCTTCCCTACAAGCGCCTACGAAAAAGAGGCTAGA
GCCATCCTGAGCAAGCAGGGCGAGTTCTACCCTGAGATCGATATCAAGT
TCGTGGAAAGCTACATTAAGATCCTGACAGATAAACGGCTGTACTATGTG
GGCCCTGGCAACGAGAAGTCTAGAACAGACTACGGCATCTACAGAGAGA
ATGGCGAAACCCTGGACAACCTGTTCGACATCCTAATCGGAAAATGTAC
CGTGTACCCCGACGAGAGAAGGGCGGCTAAATTCAGCTACACAGCCCA
GCTGTTTGATTTCCTAAACGACATGAACAACCTGTCTATCGCCGGCGAGA
AGCTGACAGAGGACCAGAAGCGAAGCATCTACCAGCAGATCATGAGCA
GCAAGACCGTGAACATGATGAAAATCATCATGAAGGTGACCGGATGTAT
CAAGGAGGATATCAAGGGCTTCAGAGTGGACAACAAGGACAAGCCTGAA
TTCCACACCTTCGAGGCTTACAGAAAGTTCCGGAACAAGATGAAGGAGG
TGAACGTGGATATCACCACCTGGTCCGACGCCGGTTTCGACGAACTGGC
TGTCCAGCTGACCTTGAGCACTGAGTACAAACAGATCGTGGAGCAGGTG
AAGAAGCTGAACCTGCTGCTGTCCGATGAGGCCATCGACGCCATCGCC
GACTTCCGGAAGAAGAATGGCACCTTGTTCTCTAAATGGCACAGTCTGT

CCCTGAAGGCCATGCACGAGATCATGGACGACCTGTGGAGCACCCATAA
GAACCAGATGCAGCTGTACACCGAACTGGGCGTCTTTAAGAGCAACAAA
GACCTGTACAAGGGACTGAAATACATCAAAAAGGAGATGCTGCTGGAAG
AAATCTACAATCCTGTGGTGAGAGGCAGCATTAGCGAGTCTATCAAGGT
GATCAACATGATGCTGAAGAAGTACGGCGCCCTGGACAAGATCGTGCTG
GAAATGCCTAGAGATGCTAATGAGAAAGAAGAAAAGAAGAGACTCGATC
AGGCCAAGAAGGATAACAAGACCCACAAGGATACAGCCATCAAAGTGGC
CCAGGAGGAATACGGCTTCAGCGAGGAAGCCTATCGGCACCACCAGGA
CCTGAATACCAAGCTGAGACTCTGGTACGAGCAGGGCAAGTGCTGTCTG
TACAGCGGCAAGACCATCAAAGTGATGGATCTGATAATGAACCCAAACC
AGTTCGACATCGACGCCATCATTCCCAAGAGCATCAGCTTCGACGACAG
CCTGAACAACAAGGTGCTCTGCTACGCAAGCGAGAACAGATGGAAGGG
CAACCAGACCCCTTTCCGGTATTTCAAGAGGAAGCAGAACAGCCAGTGG
AACTACGACAAGTATAAGTCCCAGATCGTGAAACTGTACAATGACGGCAA
GGGCAAACTGACAAGAGCCAAGATGGACCTGCTGCTGTTTGAAGAGGAC
ATCAATAAATATGAGGTTAGACGGCAGTTCATCAACAGGAACCTAGTTGA
CACCAGATACGCCAGCAGAGTGGTGCTGAACACCCTGCAAGACTTTATG
AGCGTGAACTACCCTAACACCACCGTGGGCGTGGTTAGAGGAAAGTTCA
CCGCCCAGCTGCGGAAACACTGGGGCATCAAGAAGGACCGGGACGAAT
CTCACAGCCATCACGCCATCGACGCCCTGACAGTCGCCTGCGTGCCATA
CCTGAGCCTGTGGAAGAAACAGGACATCTTCAAGGAAGAGGATATTAGA
GTGGACGATTACTCTGTGACAGTGAATGAAGAAACCGGCGAAATCATCG
AGGACAGCTACAATCAGCTGGCCTACCAGCCCCCCTTCGAGAAGTTCTT
CACAGGCCTGAGACGGGTGTCCCTCGAGTGCAGATACAGCTACAAAGTC
GACAGAAAGTTCAACCGGCAGGTGGCCGATGCCACAATCTACAGCACCC
GGCAGGGAGTGGTGAAGGAAACCAAGGGCGAGAAGAAGTTCGTGCTGG
ACCCTGATTCCAAAGAACACTACGTGGTTGCTAAGATCAAGAACATCTAT
GATGATGCTGATGCCAAGCGGTTCATGGACAGATACAAGAAGGACAAGA
CCCAGTTTCTGATGTACCACCACGACCCTCAGACCTTTGCCATCCTGGA

GGACGCCATACGGCAATATTCTAAGGAGAAGAACCCTTTCGCCATGTAC
CGGGAAGAGCACGGCCCTCTGCAAAAATACGCCAAGAAGGGCAATGGC
CCAGAGATCCGGAGCGTCAAATACTTCGACAAGAAGCTGGGAACCCACA
TCGAGCTGAAGCAGGAGCATGAGAAGAACCGCAAGGTGGTGCTGCAGA
GCCTGAACCCTTGGCGGAGCGACATCTACTACAACAAAAAAGAGAACCG
GTACATGGTGGCCGGCATCAAGTATGCAGACCTTAGCTATCAAAAGGGC
AGCGGAGAATACGGAATTTCTCTGCAGAAGTACCAGCAAGTGCTGAAGT
CAGAAAAGATCCTCCTCTCCCTGGAGGAACTGGAGGCCATCCTTAATGG
CGAGTCTACAGATAGCAATTACGAGTTCTGCTTCAGCCTGTACAAGAACA
ACCTGCTGCAGTGGACCGATGAAAAGGACCAGGTGCACCAGTACCGTTT
CCTGTCTAGAAACCTGTCTAACAGCAATATGATCGAGGTGAAGCCCATC
GAGAAATCTAAGTACGAAAAGCAGGCTTTGGGCAGAAAGACACTGACCA
AGGGCATCCACGATTTTTATAAAATCGAGGTCGACGTGCTGGGCTACCA
ATACTTCATTAAGCGGGAAAAGCTGAAGCTGTCATTCAAGTCTAGCTGCT
CCGGCGGAAGCTCTGGTGGCAGCAAGCGGACCGCCGACGGCTCTGAAT
TCGAGAGCCCTAAGAAGAAAAGAAAGGTGAGCGGAGGCTCTAGCGGCG
GAAGCACCCTGAACATTGAAGACGAGTATAGACTGCATGAAACAAGCAA
GGAACCCGACGTGTCCCTGGGCTCCACCTGGCTGTCCGACTTTCCCCA
GGCCTGGGCCGAGACAGGAGGAATGGGCCTGGCCGTGCGGCAGGCAC
CCCTGATCATCCCTCTGAAGGCCACCTCTACACCCGTGAGCATCAAGCA
GTACCCTATGTCTCAGGAGGCCAGACTGGGCATCAAGCCTCACATCCAG
AGGCTGCTGGACCAGGGCATCCTGGTGCCATGCCAGAGCCCCTGGAAC
ACACCACTGCTGCCCGTGAAGAAGCCAGGCACCAATGACTATAGACCCG
TGCAGGATCTGAGAGAGGTGAACAAGAGGGTGGAGGATATCCACCCCA
CCGTGCCCAACCCTTACAATCTGCTGTCCGGCCTGCCCCCTTCTCACCA
GTGGTATACAGTGCTGGACCTGAAGGATGCCTTCTTTTGTCTGAGACTG
CACCCTACCAGCCAGCCACTGTTCGCCTTTGAGTGGAGGGACCCTGAGA
TGGGCATCTCTGGCCAGCTGACCTGGACACGCCTGCCTCAGGGCTTCA
AGAATAGCCCAACACTGTTTAACGAGGCCCTGCACCGCGACCTGGCAGA

TTTCCGGATCCAGCACCCAGATCTGATCCTGCTGCAGTACGTGGACGAT
CTGCTGCTGGCCGCCACCAGCGAGCTGGATTGCCAGCAGGGAACACGC
GCCCTGCTGCAGACCCTGGGAAACCTGGGATATAGGGCATCCGCCAAG
AAGGCCCAGATCTGTCAGAAGCAGGTGAAGTACCTGGGCTATCTGCTGA
AGGAGGGCCAGAGATGGCTGACAGAGGCCAGGAAGGAGACAGTGATG
GGCCAGCCAACACCCAAGACCCCAAGACAGCTGAGGGAGTTCCTGGGC
AAAGCAGGATTTTGCAGGCTGTTCATCCCAGGATTCGCAGAGATGGCAG
CACCTCTGTACCCACTGACCAAGCCGGGCACCCTGTTTAATTGGGGCCC
TGACCAGCAGAAGGCCTATCAGGAGATCAAGCAGGCCCTGCTGACAGC
ACCAGCCCTGGGCCTGCCAGACCTGACCAAGCCTTTCGAGCTGTTTGTG
GATGAGAAGCAGGGCTACGCCAAGGGCGTGCTGACCCAGAAGCTGGGA
CCATGGAGACGGCCCGTGGCCTATCTGTCCAAGAAGCTGGACCCAGTG
GCAGCAGGATGGCCACCATGCCTGAGGATGGTGGCAGCAATCGCCGTG
CTGACAAAGGATGCCGGCAAGCTGACCATGGGACAGCCACTGGTCATC
CTGGCACCACACGCAGTGGAGGCCCTGGTGAAGCAGCCTCCAGATCGC
TGGCTGTCTAACGCCCGGATGACACACTACCAGGCCCTGCTGCTGGACA
CCGATCGCGTGCAGTTTGGCCCTGTGGTGGCCCTGAATCCAGCCACCC
TGCTGCCTCTGCCAGAGGAGGGCCTGCAGCACAACTGTCTGGACTCTG
GCGGCTCAAAAAGAACCGCCGACGGCAGCGAATTCGAGCCCAAGAAGA
AGAGGAAAGTCGGCTCTGGCCCTGCCGCTAAGAGAGTGAAGCTGGACG
GATCCGGCGAGGGCAGAGGAAGTCTGCTAACATGCGGTGACGTCGAGG
AGAATCCTGGCCCAGTGAGCAAGGGCGAGGAGCTGTTCACCGGGGTGG
TGCCCATCCTGGTCGAGCTGGACGGCGACGTAAACGGCCACAAGTTCA
GCGTGTCCGGCGAGGGCGAGGGCGATGCCACCTACGGCAAGCTGACC
CTGAAGTTCATCTGCACCACCGGCAAGCTGCCCGTGCCCTGGCCCACC
CTCGTGACCACCCTGACCTACGGCGTGCAGTGCTTCAGCCGCTACCCC
GACCACATGAAGCAGCACGACTTCTTCAAGTCCGCCATGCCCGAAGGCT
ACGTCCAGGAGCGCACCATCTTCTTCAAGGACGACGGCAACTACAAGAC
CCGCGCCGAGGTGAAGTTCGAGGGCGACACCCTGGTGAACCGCATCGA

GCTGAAGGGCATCGACTTCAAGGAGGACGGCAACATCCTGGGGCACAA
GCTGGAGTACAACTACAACAGCCACAACGTCTATATCATGGCCGACAAG
CAGAAGAACGGCATCAAGGTGAACTTCAAGATCCGCCACAACATCGAGG
ACGGCAGCGTGCAGCTCGCCGACCACTACCAGCAGAACACCCCCATCG
GCGACGGCCCCGTGCTGCTGCCCGACAACCACTACCTGAGCACCCAGT
CCGCCCTGAGCAAAGACCCCAACGAGAAGCGCGATCACATGGTCCTGC
TGGAGTTCGTGACCGCCGCCGGGATCACTCTCGGCATGGACGAGCTGT
ACAAGTAA

Sequences S4. Sequences of SpCas9-NG-PE6d-T2A-GFP

NLS-SpCas9-NG(H840A)-Linker- MMLV RT∆RNaseH(T128N, V223Y, D200C)-NLS-T2A-GFP

CCAAAGAAGAAGCGGAAAGTCGACAAGAAGTACAGCATCGGCCTGGAC
ATCGGCACCAACTCTGTGGGCTGGGCCGTGATCACCGACGAGTACAAG
GTGCCCAGCAAGAAATTCAAGGTGCTGGGCAACACCGACCGGCACAGC
ATCAAGAAGAACCTGATCGGAGCCCTGCTGTTCGACAGCGGCGAAACAG
CCGAGGCCACCCGGCTGAAGAGAACCGCCAGAAGAAGATACACCAGAC
GGAAGAACCGGATCTGCTATCTGCAAGAGATCTTCAGCAACGAGATGGC
CAAGGTGGACGACAGCTTCTTCCACAGACTGGAAGAGTCCTTCCTGGTG
GAAGAGGATAAGAAGCACGAGCGGCACCCCATCTTCGGCAACATCGTG
GACGAGGTGGCCTACCACGAGAAGTACCCCACCATCTACCACCTGAGAA
AGAAACTGGTGGACAGCACCGACAAGGCCGACCTGCGGCTGATCTATCT
GGCCCTGGCCCACATGATCAAGTTCCGGGGCCACTTCCTGATCGAGGG
CGACCTGAACCCCGACAACAGCGACGTGGACAAGCTGTTCATCCAGCTG
GTGCAGACCTACAACCAGCTGTTCGAGGAAAACCCCATCAACGCCAGCG
GCGTGGACGCCAAGGCCATCCTGTCTGCCAGACTGAGCAAGAGCAGAC

GGCTGGAAAATCTGATCGCCCAGCTGCCCGGCGAGAAGAAGAATGGCC

TGTTCGGAAACCTGATTGCCCTGAGCCTGGGCCTGACCCCCAACTTCAA

GAGCAACTTCGACCTGGCCGAGGATGCCAAACTGCAGCTGAGCAAGGA

CACCTACGACGACGACCTGGACAACCTGCTGGCCCAGATCGGCGACCA

GTACGCCGACCTGTTTCTGGCCGCCAAGAACCTGTCCGACGCCATCCTG

CTGAGCGACATCCTGAGAGTGAACACCGAGATCACCAAGGCCCCCCTG

AGCGCCTCTATGATCAAGAGATACGACGAGCACCACCAGGACCTGACCC

TGCTGAAAGCTCTCGTGCGGCAGCAGCTGCCTGAGAAGTACAAAGAGAT

TTTCTTCGACCAGAGCAAGAACGGCTACGCCGGCTACATTGACGGCGGA

GCCAGCCAGGAAGAGTTCTACAAGTTCATCAAGCCCATCCTGGAAAAGA

TGGACGGCACCGAGGAACTGCTCGTGAAGCTGAACAGAGAGGACCTGC

TGCGGAAGCAGCGGACCTTCGACAACGGCAGCATCCCCCACCAGATCC

ACCTGGGAGAGCTGCACGCCATTCTGCGGCGGCAGGAAGATTTTTACCC

ATTCCTGAAGGACAACCGGGAAAAGATCGAGAAGATCCTGACCTTCCGC

ATCCCCTACTACGTGGGCCCTCTGGCCAGGGGAAACAGCAGATTCGCCT

GGATGACCAGAAAGAGCGAGGAAACCATCACCCCCTGGAACTTCGAGG

AAGTGGTGGACAAGGGCGCTTCCGCCCAGAGCTTCATCGAGCGGATGA

CCAACTTCGATAAGAACCTGCCCAACGAGAAGGTGCTGCCCAAGCACAG

CCTGCTGTACGAGTACTTCACCGTGTATAACGAGCTGACCAAAGTGAAAT

ACGTGACCGAGGGAATGAGAAAGCCCGCCTTCCTGAGCGGCGAGCAGA

AAAAGGCCATCGTGGACCTGCTGTTCAAGACCAACCGGAAAGTGACCGT

GAAGCAGCTGAAAGAGGACTACTTCAAGAAAATCGAGTGCTTCGACTCC

GTGGAAATCTCCGGCGTGGAAGATCGGTTCAACGCCTCCCTGGGCACAT

ACCACGATCTGCTGAAAATTATCAAGGACAAGGACTTCCTGGACAATGA

GGAAAACGAGGACATTCTGGAAGATATCGTGCTGACCCTGACACTGTTT

GAGGACAGAGAGATGATCGAGGAACGGCTGAAAACCTATGCCCACCTGT

TCGACGACAAAGTGATGAAGCAGCTGAAGCGGCGGAGATACACCGGCT

GGGGCAGGCTGAGCCGGAAGCTGATCAACGGCATCCGGGACAAGCAGT

CCGGCAAGACAATCCTGGATTTCCTGAAGTCCGACGGCTTCGCCAACAG

AAACTTCATGCAGCTGATCCACGACGACAGCCTGACCTTTAAAGAGGAC
ATCCAGAAAGCCCAGGTGTCCGGCCAGGGCGATAGCCTGCACGAGCAC
ATTGCCAATCTGGCCGGCAGCCCCGCCATTAAGAAGGGCATCCTGCAGA
CAGTGAAGGTGGTGGACGAGCTCGTGAAAGTGATGGGCCGGCACAAGC
CCGAGAACATCGTGATCGAAATGGCCAGAGAGAACCAGACCACCCAGAA
GGGACAGAAGAACAGCCGCGAGAGAATGAAGCGGATCGAAGAGGGCAT
CAAAGAGCTGGGCAGCCAGATCCTGAAAGAACACCCCGTGGAAAACAC
CCAGCTGCAGAACGAGAAGCTGTACCTGTACTACCTGCAGAATGGGCG
GGATATGTACGTGGACCAGGAACTGGACATCAACCGGCTGTCCGACTAC
GATGTGGACGCCATCGTGCCTCAGAGCTTTCTGAAGGACGACTCCATCG
ACAACAAGGTGCTGACCAGAAGCGACAAGAACCGGGGCAAGAGCGACA
ACGTGCCCTCCGAAGAGGTCGTGAAGAAGATGAAGAACTACTGGCGGC
AGCTGCTGAACGCCAAGCTGATTACCCAGAGAAAGTTCGACAATCTGAC
CAAGGCCGAGAGAGGCGGCCTGAGCGAACTGGATAAGGCCGGCTTCAT
CAAGAGACAGCTGGTGGAAACCCGGCAGATCACAAAGCACGTGGCACA
GATCCTGGACTCCCGGATGAACACTAAGTACGACGAGAATGACAAGCTG
ATCCGGGAAGTGAAAGTGATCACCCTGAAGTCCAAGCTGGTGTCCGATT
TCCGGAAGGATTTCCAGTTTTACAAAGTGCGCGAGATCAACAACTACCAC
CACGCCCACGACGCCTACCTGAACGCCGTCGTGGGAACCGCCCTGATC
AAAAAGTACCCTAAGCTGGAAAGCGAGTTCGTGTACGGCGACTACAAGG
TGTACGACGTGCGGAAGATGATCGCCAAGAGCGAGCAGGAAATCGGCA
AGGCTACCGCCAAGTACTTCTTCTACAGCAACATCATGAACTTTTTCAAG
ACCGAGATTACCCTGGCCAACGGCGAGATCCGGAAGCGGCCTCTGATC
GAGACAAACGGCGAAACCGGGGAGATCGTGTGGGATAAGGGCCGGGAT
TTTGCCACCGTGCGGAAAGTGCTGAGCATGCCCCAAGTGAATATCGTGA
AAAAGACCGAGGTGCAGACAGGCGGCTTCAGCAAAGAGTCTATCAGGC
CCAAGAGGAACAGCGATAAGCTGATCGCCAGAAAGAAGGACTGGGACC
CTAAGAAGTACGGCGGCTTCGTCAGCCCCACCGTGGCCTATTCTGTGCT
GGTGGTGGCCAAAGTGGAAAAGGGCAAGTCCAAGAAACTGAAGAGTGT

GAAAGAGCTGCTGGGGATCACCATCATGGAAAGAAGCAGCTTCGAGAAG
AATCCCATCGACTTTCTGGAAGCCAAGGGCTACAAAGAAGTGAAAAAGG
ACCTGATCATCAAGCTGCCTAAGTACTCCCTGTTCGAGCTGGAAAACGG
CCGGAAGAGAATGCTGGCCTCTGCCCGCTTCCTGCAGAAGGGAAACGA
ACTGGCCCTGCCCTCCAAATATGTGAACTTCCTGTACCTGGCCAGCCAC
TATGAGAAGCTGAAGGGCTCCCCCGAGGATAATGAGCAGAAACAGCTGT
TTGTGGAACAGCACAAGCACTACCTGGACGAGATCATCGAGCAGATCAG
CGAGTTCTCCAAGAGAGTGATCCTGGCCGACGCTAATCTGGACAAAGTG
CTGTCCGCCTACAACAAGCACCGGGATAAGCCCATCAGAGAGCAGGCC
GAGAATATCATCCACCTGTTTACCCTGACCAATCTGGGAGCCCCTCGCG
CCTTCAAGTACTTTGACACCACCATCGACCGGAAGGTGTACAGAAGCAC
CAAAGAGGTGCTGGACGCCACCCTGATCCACCAGAGCATCACCGGCCT
GTACGAGACACGGATCGACCTGTCTCAGCTGGGAGGCGACTCCGGCGG
AAGCTCTGGTGGCAGCAAGCGGACCGCCGACGGCTCTGAATTCGAGAG
CCCTAAGAAGAAAAGAAAGGTGAGCGGAGGCTCTAGCGGCGGAAGC
ACCCTGAACATTGAAGACGAGTATAGACTGCATGAAACAAGCAAGGAAC
CCGACGTGTCCCTGGGCTCCACCTGGCTGTCCGACTTTCCCCAGGCCT
GGGCCGAGACAGGAGGAATGGGCCTGGCCGTGCGGCAGGCACCCCTG
ATCATCCCTCTGAAGGCCACCTCTACACCCGTGAGCATCAAGCAGTACC
CTATGTCTCAGGAGGCCAGACTGGGCATCAAGCCTCACATCCAGAGGCT
GCTGGACCAGGGCATCCTGGTGCCATGCCAGAGCCCCTGGAACACACC
ACTGCTGCCCGTGAAGAAGCCAGGCACCAATGACTATAGACCCGTGCAG
GATCTGAGAGAGGTGAACAAGAGGGTGGAGGATATCCACCCCACCGTG
CCCAACCCTTACAATCTGCTGTCCGGCCTGCCCCCTTCTCACCAGTGGT
ATACAGTGCTGGACCTGAAGGATGCCTTCTTTTGTCTGAGACTGCACCCT
ACCAGCCAGCCACTGTTCGCCTTTGAGTGGAGGGACCCTGAGATGGGC
ATCTCTGGCCAGCTGACCTGGACACGCCTGCCTCAGGGCTTCAAGAATA
GCCCAACACTGTTTAACGAGGCCCTGCACCGCGACCTGGCAGATTTCCG
GATCCAGCACCCAGATCTGATCCTGCTGCAGTACGTGGACGATCTGCTG

CTGGCCGCCACCAGCGAGCTGGATTGCCAGCAGGGAACACGCGCCCTG
CTGCAGACCCTGGGAAACCTGGGATATAGGGCATCCGCCAAGAAGGCC
CAGATCTGTCAGAAGCAGGTGAAGTACCTGGGCTATCTGCTGAAGGAGG
GCCAGAGATGGCTGACAGAGGCCAGGAAGGAGACAGTGATGGGCCAGC
CAACACCCAAGACCCCAAGACAGCTGAGGGAGTTCCTGGGCAAAGCAG
GATTTTGCAGGCTGTTCATCCCAGGATTCGCAGAGATGGCAGCACCTCT
GTACCCACTGACCAAGCCGGGCACCCTGTTTAATTGGGGCCCTGACCAG
CAGAAGGCCTATCAGGAGATCAAGCAGGCCCTGCTGACAGCACCAGCC
CTGGGCCTGCCAGACCTGACCAAGCCTTTCGAGCTGTTTGTGGATGAGA
AGCAGGGCTACGCCAAGGGCGTGCTGACCCAGAAGCTGGGACCATGGA
GACGGCCCGTGGCCTATCTGTCCAAGAAGCTGGACCCAGTGGCAGCAG
GATGGCCACCATGCCTGAGGATGGTGGCAGCAATCGCCGTGCTGACAA
AGGATGCCGGCAAGCTGACCATGGGACAGCCACTGGTCATCCTGGCAC
CACACGCAGTGGAGGCCCTGGTGAAGCAGCCTCCAGATCGCTGGCTGT
CTAACGCCCGGATGACACACTACCAGGCCCTGCTGCTGGACACCGATC
GCGTGCAGTTTGGCCCTGTGGTGGCCCTGAATCCAGCCACCCTGCTGC
CTCTGCCAGAGGAGGGCCTGCAGCACAACTGTCTGGACTCTGGCGGCT
CAAAAAGAACCGCCGACGGCAGCGAATTCGAGCCCAAGAAGAAGAGGA
AAGTCGGCTCTGGCCCTGCCGCTAAGAGAGTGAAGCTGGACGGATCCG
GCGAGGGCAGAGGAAGTCTGCTAACATGCGGTGACGTCGAGGAGAATC
CTGGCCCAGTGAGCAAGGGCGAGGAGCTGTTCACCGGGGTGGTGCCCA
TCCTGGTCGAGCTGGACGGCGACGTAAACGGCCACAAGTTCAGCGTGT
CCGGCGAGGGCGAGGGCGATGCCACCTACGGCAAGCTGACCCTGAAGT
TCATCTGCACCACCGGCAAGCTGCCCGTGCCCTGGCCCACCCTCGTGA
CCACCCTGACCTACGGCGTGCAGTGCTTCAGCCGCTACCCCGACCACAT
GAAGCAGCACGACTTCTTCAAGTCCGCCATGCCCGAAGGCTACGTCCAG
GAGCGCACCATCTTCTTCAAGGACGACGGCAACTACAAGACCCGCGCC
GAGGTGAAGTTCGAGGGCGACACCCTGGTGAACCGCATCGAGCTGAAG
GGCATCGACTTCAAGGAGGACGGCAACATCCTGGGGCACAAGCTGGAG

TACAACTACAACAGCCACAACGTCTATATCATGGCCGACAAGCAGAAGA
ACGGCATCAAGGTGAACTTCAAGATCCGCCACAACATCGAGGACGGCAG
CGTGCAGCTCGCCGACCACTACCAGCAGAACACCCCCATCGGCGACGG
CCCCGTGCTGCTGCCCGACAACCACTACCTGAGCACCCAGTCCGCCCT
GAGCAAAGACCCCAACGAGAAGCGCGATCACATGGTCCTGCTGGAGTT
CGTGACCGCCGCCGGGATCACTCTCGGCATGGACGAGCTGTACAAGTA
A
